# Modern Chemical Routes for the Controlled Synthesis of Anisotropic Bimetallic Nanostructures and Their Application in Catalysis

**DOI:** 10.3389/fchem.2020.00357

**Published:** 2020-05-19

**Authors:** Prangya Bhol, M. B. Bhavya, Swarnalata Swain, Manav Saxena, Akshaya K. Samal

**Affiliations:** Centre for Nano and Material Sciences, Jain Global Campus, Jain University, Ramanagara, India

**Keywords:** anisotropic nanostructures, galvanic reaction, antigalvanic reaction, bimetallic nanostructures, catalysis

## Abstract

Bimetallic nanoparticles (BNPs) have attracted greater attention compared to its monometallic counterpart because of their chemical/physical properties. The BNPs have a wide range of applications in the fields of health, energy, water, and environment. These properties could be tuned with a number of parameters such as compositions of the bimetallic systems, their preparation method, and morphology. Monodisperse and anisotropic BNPs have gained considerable interest and numerous efforts have been made for the controlled synthesis of bimetallic nanostructures (BNS) of different sizes and shapes. This review offers a brief summary of the various synthetic routes adopted for the synthesis of Palladium(Pd), Platinum(Pt), Nickel(Ni), Gold(Au), Silver(Ag), Iron(Fe), Cobalt(Co), Rhodium(Rh), and Copper(Cu) based transition metal bimetallic anisotropic nanostructures, growth mechanisms e.g., seed mediated co-reduction, hydrothermal, galvanic replacement reactions, and antigalvanic reaction, and their application in the field of catalysis. The effect of surfactant, reducing agent, metal precursors ratio, pH, and reaction temperature for the synthesis of anisotropic nanostructures has been explained with examples. This review further discusses how slight modifications in one of the parameters could alter the growth mechanism, resulting in different anisotropic nanostructures which highly influence the catalytic activity. The progress or modification implied in the synthesis techniques within recent years is focused on in this article. Furthermore, this article discussed the improved activity, stability, and catalytic performance of BNS compared to the monometallic performance. The synthetic strategies reported here established a deeper understanding of the mechanisms and development of sophisticated and controlled BNS for widespread application.

## Introduction

Bimetallic nanostructures (BNS) have gained worldwide attention due to the enhanced properties they have compared to its counterparts and have widely been explored in different applications (Sharma et al., 2017). In the early 1980s the words “bimetallic cluster” were first introduced by John H. Sinfelt during his series of research over bimetallic nanocatalysts supported on silica or alumina (Sinfelt et al., 1980, 1981, 1982). BNS were synthesized by combining two different metals where the constituent metals either form an alloy or a nanocomposite including core shell and nanodendrites (Tao, 2012). The presence of two or more metals with their respective nanometric shape and size decides the development of novel properties from its pristine form. Moreover, the formation of self-accumulation structures has been gaining significant interest and suggests attractive options for the synthesis of bimetallic nanomaterials with unique functions (Rechberger and Niederberger, 2017). Unique properties developed in a single material from the manifestation of multiple metals can be used in different applications such as catalysis, sensing, thermoelectric, and electronic devices (Barakat et al., 2010; Alonso et al., 2012; Piccolo, 2012; Tao, 2012; Liu and Astruc, 2017). Diverse routes have been adapted for the synthesis of bimetallic nanoparticles, regardless of the engineering of these bimetallic structures with monodispersity. Controlled shape and size in bulk is the most important challenge.

Why are anisotropic nanomaterials with monodispersity in incessant demand? When anisotropy is introduced to any nanoparticle for various shapes, sizes, and monodisperse morphologies (such as rods, cubes, octahedrons, dodecahedrons, triangles, etc.) different facets with different surface energies are generated, producing novel physical and chemical properties (Sajanlal et al., 2011). In addition to that, two or more metals forming anisotropic nanostructures such as core-shells, alloys, or complexes alter the atomic and electronic arrangements in them and attribute better characteristic performance (Alonso et al., 2012; Tao, 2012; Ruditskiy et al., 2016; Tao et al., 2016). Moreover, the shape of the nanomaterial also affects the motion of electrons, holes, plasmons, and excitons according to its directional growth and hence generates different properties. For example, in the case of an isotropic nanoparticle (i.e., a sphere) the electrons are confined symmetrically in all directions and, hence, the properties will be more or less same in all direction. Whereas in the case of anisotropic nanomaterials, which are further classified as 1D, 2D, and 3D nanostructures, further directional dependent physical and chemical properties are posed because of the available motion of electron in these dimensions (Sajanlal et al., 2011). The facets possess a disproportionate number of atoms or molecules over the surface which influence the reactivity, catalytic activity, resistance, and optoelectronics properties. Different facets play a considerable role in enhancing the catalytic activity or efficiency because the atoms present in different planes, edges, or corners possess different surface energies and thus different reactivities (Mahmoud et al., 2013; Mahmoud and El-Sayed, 2014). The sharp edge present in the corner shows improved reactivity in comparison to the atoms present in other sites or in isotropic particles (Tao et al., 2008). Further alteration of the elemental composition in the synthesis of complex nanocatalysts opens up new dimensions due to the development of new facets and electronic properties. Enhanced properties are achieved from bimetallic nanocatalysts than from its monometallic counterparts (Wang et al., 2011). BNS have been widely explored in the combination of rare elements such as Au, Pd, and Pt with cheaper metals like Ni, Cu, and Fe for enhanced catalytic activity, selectivity, high recovery, and large-scale industrial applications due to its development of novel characteristics.

Additionally, bimetallic nanocatalysts have shown effective catalytic behavior and an enriched stability and activity compared to monometallic NPs (Mahmoud and El-Sayed, 2014). BNPs have been synthesized in various shapes, sizes, and structures using different protocols (Yuan et al., 2015). The reaction environment plays a key role in establishing the structure and property of the multiple metals participating in the reaction (Santana et al., 2018). The general method to synthesize bimetallic nanostructure involves the successive reduction of metal ions providing an appropriate stabilizing agent in order to avoid agglomeration as well as preferential growth of the nanostructure. The traditional synthesis method for bimetallic nanostructure uses template and surfactant processes (Ahmad et al., 2019), sol-gel methods (Gołabiewska et al., 2017), and electrochemical depositions (Vilana et al., 2015). These methods require the removal of the template, surfactant, and substrate for the purification of product. Moreover, certain reactions have been conducted at elevated temperatures and have resulted in large unalloyed NPs, lowered the probability of any interaction of the two metals, and provided their respective characteristics (Mallikarjuna et al., 2018). To overcome these drawbacks, many advanced or modified methods have been proposed for the synthesis of BNS where the interaction of two metals provides high stability, enhanced activity, and unique electrical and chemical properties. The recent discovery of an antigalvanic reaction (AGR) which contradicts the galvanic principle has attracted much attention for its synthesis, mechanism, and catalytic applications (Gan et al., 2018). Transition metal NPs such as Pd, Pt, Ni, and Cu have been widely used due to the easy synthetic protocol and have a wide range of applications in the field of catalysis.

Here, we have discussed most of the remarkable anisotropic BNS such as cubic nanoframes (Ye et al., 2015), nanoboxes (Ye et al., 2015), nanocubes (Lim et al., 2010), nanorods (Sun et al., 2017), octopods (Kunz et al., 2017), and trigonal nanoframes (Chen et al., 2015) synthesized by different techniques such as seed mediated method (Kunz et al., 2017), galvanic method (González et al., 2011), hydrothermal method (Wu et al., 2019b), and electrospinning technique (Ghouri et al., 2015). The major objective of this article is to discuss the updated synthesis mechanisms as well as to provide a better understanding of the factors responsible for generating complex bimetallic anisotropic nanostructures with high monodispersity. In addition to that, the discussion highlights the importance of bimetallic properties in the field of catalysis, photocatalysis, and electrocatalysis. Highly dispersed anisotropic BNS offers a fascinating path to design new catalysts with novel properties and reactivity with respect to different facets. In order to understand the concept, the discussion starts gradually with the history and the revolution in the synthetic approaches to achieve monodispersed anisotropic bimetallic nanostructures. The discussion is further exemplified with a few illustrations of different techniques giving information about factors such as precursor's ratio, reaction pH, temperature, and capping agents responsible in engineering the final morphology and internal composition. The review further explicitly highlights galvanic replacement reactions mechanisms and its uses to develop complex BNS. New approaches adapted through a combination with other mechanisms like Kirkendall, sequential deposition, and co-reduction have been explained with examples.

### Revolution of Synthetic Methods

**Figure 1:**
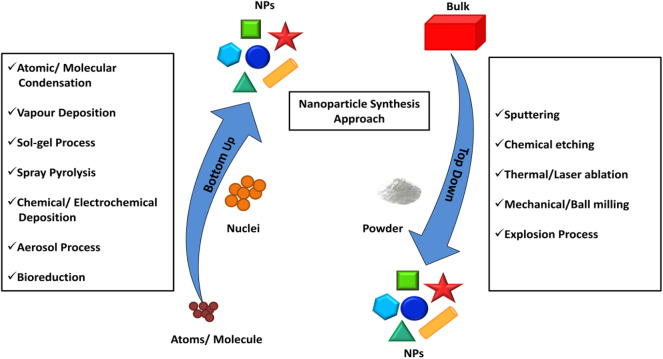
Top–down and bottom–up approaches exploiting different physical, chemical, and biological methods for the synthesis of NPs. Redrawn from the data in Ovais et al. (2017).

A significant amount of knowledge has been gathered on the synthetic approaches of BNS over the last few decades. The general strategy to synthesize nanomaterial is either “top-down methods” or “bottom-up methods” as shown in Figure 1 (Ovais et al., 2017). The top-down method starts with a bulk structure and then it reduces to nanoscale via an etching out of the crystal planes of the initial structure. In a general way it could be said that the building blocks are removed from the bulk to shape it into desired nanostructures. This process involves physical processes such as the mechanical grinding or ball milling methods that grins the bulk material into a fine powder with the selective addition of stabilizing agents to prevent agglomeration and result in a nanomaterial (Yadav et al., 2012). Although these mechanical methods provide the least expensive paths to synthesize nanomaterials, where kinetic energy was developed during the breaking up of the bulk structure is useful in the reduction of materials. This method fails to synthesize fine anisotropic nanostructures with high monodispersity.

The breaking of bulk often results in uneven surfaces or ruptured edges in the nanomaterial which can further impact on the physiochemical property as well as the surface energy of the BNS. Bottom up method resolves these issues and provides a fascinating path to designing monodispersed anisotropic nanostructures. In this method, the nanostructures are assembled from the bottom by stacking atom-by-atom, molecule-by-molecule resulting in a uniform distribution of building blocks. The method starts from the reduction of precursors to atoms followed by nucleation and growth process.

In the year 1857, Michael Faraday was the first person to propose the general method of chemical reduction of gold and other metal salts in the presence of a suitable stabilizer to synthesize a metal nanoparticle dispersed in an aqueous solvent, which turned out to be the most powerful and common technique in the synthesis field (Faraday, 1857). After his discovery, Turkevich carried out several experiments and established a standard synthesis protocol where he successfully synthesized Pt (Aika et al., 1976), Au (Turkevich et al., 1951) and Pd (Turkevich and Kim, 1970) nanoparticles using sodium citrate as the reducing agent for the supported catalyst. He concluded that the mechanism involves successive nucleation, growth, and agglomeration forming nanoparticles. Hence, the rate of nucleation and growth of the cluster determines the size of the particle. With passing time, the research into bimetallic nanoparticle generated great interest. A series of studies carried out on bimetallic nanoparticles as catalysts over silica such as Ru-Cu (Sinfelt et al., 1980), Os-Cu (Sinfelt et al., 1981), and Pt-Ir (Sinfelt et al., 1982) by Sinfelt et al. over bimetallic nanostructures influencing the catalytic activity. The morphology of bimetallic nanostructures was analyzed using X-ray absorption fine structure (EXAFS) technique and confirmed the spherical structure of BNPs with homogeneous distribution of two metals forming an alloy (Meitzner et al., 1983, 1985). BNPs synthesis involves either co-reduction or successive reduction of two metal precursors. The co-reduction method is the simplest approach to synthesizing bimetallic nanoparticles and is similar to monometallic nanoparticle synthesis, where successive reduction of two metals is followed in order to synthesize core-shell bimetallic nanostructure. The efforts of countless researchers have made tight control over the morphology and composition of BNS possible.

Bimetallic nanoparticles such as Au-Pt or Pd-Pt are synthesized using sodium citrate as a reducing agent (Harada et al., 1992). Polymer-stabilized bimetallic nanoparticles in a water–alcohol system was also studied (Toshima and Wang, 1993, 1994a,b; Schmidt et al., 1997). The synthesis of Pd-Pt bimetallic nanoparticles under the simultaneous reduction of two metal precursors i.e. palladium (II) chloride and hexachloro-platinic (IV) acid was carried out using a stabilizing agent, poly(N-vinyl-2-pyrrolidone) (PVP) in continuous reflux of ethanol-water (1:1 v/v). The synthesis of light transition metals nanoparticles is difficult because of its lower redox potentials. But light transition metal nanoparticles are very important in the field of catalysis and as magnetic materials. These are prepared with the modified alcohol reduction method with additional treatment of several other chemical components like alcohol, glucose, NaOH, and tetraoctylammoniumbromide with a polymer which influences the synthesis of bimetallic nanoparticles.

Thermal decomposition in the presence of PVP was reported by Bradley with a certain modification of method (Esumi et al., 1990; Bradley et al., 1993). In the reaction, heating of palladium and copper acetates in the presence of a high boiling point solvent, ethoxyethanol, produces a PVP-stabilized Pd-Cu nanoparticle. Surfactant assisted bimetallic nanoparticles of gold/palladium using ultrasonic irradiation was reported by Mizukoshi and co, which later was termed the sonochemical method (Mizukoshi et al., 1997). In the synthesis, the surfactant, sodium dodecyl sulfate (SDS), was used to stabilize the reaction where constant ultrasonic irradiation reduces the Au(III) and Pd(II) ions in the aqueous medium. Radiolysis synthesis is another highly exploited synthetic method from the 1990s where numerous reports confirmed the synthesis of bimetallic by γ radiolysis. The method was mostly used to synthesize bimetallic of noble metals [for example, Ag-Pt BNPs prepared by radiolysis of the aqueous mixture of Ag_2_SO_4_ and K_2_PtCl_4_ (Remita et al., 1996)]. Similarly, γ radiolysis was used to synthesize Ag-Au bimetallic nanoparticles with an Au layer over an Ag nanoparticle (Henglein, 1993). Most of the techniques discussed above include the early efforts of researchers in order to design reproducible methods for synthesizing BNPs with the reduction of metal ions in the aqueous medium with suitable treatment of a stabilizing agent.

With the help of reducing agents such as trisodium citrate, hydrazine hydrate, ascorbic acid, alcohols, carbon monoxide, LiAlH_4_, and NaBH_4_, the metal ions are reduced to their nanometric size and shape (Service, 2007; Armbrster et al., 2010). The evolution of each technique was accomplished with modifications in the past reports and focused on the monodispersed controlled synthesis of bimetallic nanoparticles. The successful synthesis of BNS encourages researchers to synthesize more complex multimetallic nanostructures. Selective element segregation, diffusion, and electrochemical etching of metals have been employed to synthesize desired multimetallic nanostructures (Saleem et al., 2019). The monodispersity in the nanostructure offers facet dependence physiochemical properties which can highly influence the catalytic performance. Several attempts were done by adapting control reaction conditions to get fine monodispersity in the nanostructures.

## Synthetic Methods

### Seed Mediated Growth Technique

Seed mediated growth mechanism is one of the most common and traditional techniques for the synthesis of anisotropic bimetallic nanostructures. As bimetallic nanostructures involve two metal precursors, the first step is the reduction of one metal using a preferential choice of reducing agent and surfactant to form seeds. Then, the other metal precursor is added in the presence of the seed with surfactant and reducing agent for the controlled overgrowth of the second metal on the well-defined surface of the seeds of the first metals. Physical parameters have played a major role in the structural growth of the BNS. Lattice match, correlation of surface interfacial energies, electro negativities between the two metals, and reduction potentials are the crucial factors which are responsible for the nucleation and growth of bimetallic nanocrystals (Mahmoud and El-Sayed, 2014). Reducing and capping agents have also been considered as vital factors that govern the growth of nanostructures. The reduction potential of the two metals in bimetallic determine the formation of an alloy or core-shell. Metals having very close reduction potentials follow the seed mediated co-reduction (SMCR) technique and reduce together to form an alloy. If two metals have a significant difference in their reduction potentials, they form a preferentially core-shell structure. Pd-Au bimetallic nanocubes (Lim et al., 2010), Pd-Cu diverse nanostructures (Kunz et al., 2017), Au-Pd bimetallic nanorods (Sun et al., 2017), and Au-Pd nano rings (Wang W. et al., 2016) were some examples synthesized using the seed mediated method.

#### Nanocubes

**Figure 2:**
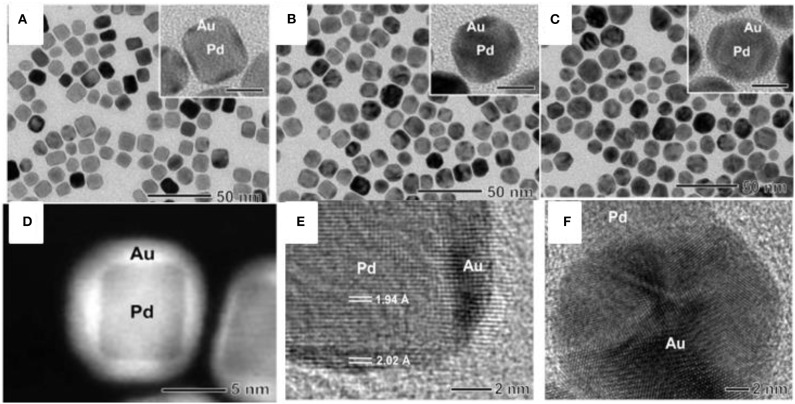
**(A–C)** TEM images of Pd-Au core-shell nanocrystals at different concentrations of HAuCl_4_ **(A)** 0.53, **(B)** 0.80, and **(C)** 1.33 mM. **(D)** HAADF-STEM image of Pd-Au core-shell nanocrystals. **(E)** HRTEM image of one of the Pd-Au core-shell nanocrystals. **(F)** HRTEM image of a Pd-Au dimer, showing the partial decahedral structure of an Au particle attached to a Pd nanocube [Reprinted with permission from Lim et al. (2010), Copyright 2010, American Chemical Society].

Au and Pd have been investigated more extensively compared to other BNS owing to their excellent catalytic performance compared to their monometallic counterparts. Both Au and Pd have a face-centered cubic (fcc) crystalline arrangement with slight mismatch (~4%) and can form materials with a wide range of stoichiometry which provides a path to designing diverse Au-Pd nanostructures. Regardless of differences in their redox potential they may reduce together to form alloys via controlling the diffusion environment where the surface deposition occurs faster than diffusion of reactants to the surface of nucleus. Pd-Au core-shell nanocrystals are synthesized using seed mediated method reported elsewhere (Lim et al., 2010). The thickness of the Au shell depends on the concentration of a gold (III) chloride (HAuCl_4_) precursor. Initially, Pd nanocube seed was synthesized using L-ascorbic acid (AA) and bromide ions as the reducing and capping agents, respectively, and favors the growth in (100) direction. Further, the accumulation of Au on the surface of cubic Pd seeds by the reduction of HAuCl_4_ in presence of mild reducing agent, AA was carried out. Different concentrations of HAuCl_4_ were added to Pd nanocubes for the controlled overgrowth of different thicknesses of Au on Pd as shown in Figures 2A–C. TEM image confirmed the overgrowth of Au as a shell of thickness 1–2 nm on the Pd nanocubes. The shape of the Pd core retained its original cubic shape, indicating that there is no loss of Pd during the accumulation of an Au shell.

The shell thickness of Au on Pd is dependent on different concentrations of HAuCl_4_, forming a core-shell. Upon increasing the concentration of HAuCl_4_, the thickness of Au shells increased 3–5 nm accordingly on Pd cubes without altering the shape of the Pd core as shown in Figure 2A. A bimetallic core-shell nanostructure was clearly seen in a high-angle annular dark-field scanning transmission electron microscopy (HAADF-STEM) image as shown in Figure 2D. This can be seen due to the contrast between the Pd and Au metals. At a low concentration of HAuCl_4_, the thinner shell of Au coated over all facets of the Pd nanocube. The high-resolution transmission electron microscopy (HRTEM) image in Figure 2E shows the inter planar distance of Pd and Au indicated an epitaxial growth of Au on Pd. Figure 2F shows the high resolution TEM image of Pd-Au dimer, the Au develops with partial decahedral structure over Pd nanocube.

#### Nanooctopods

The seed mediated co-reduction (SMCR) technique is used to synthesize Pd-Cu nanostructures of larger lattice mismatch (~7%) between precursor metals (Kunz et al., 2017). In this technique, a variety of alloy structures are synthesized due to the co-deposition of Pd and Cu on top of cubic Pd seeds. Pd nanocube seed was prepared by mixing a Pd precursor with cetyltrimethylammonium bromide (CTAB) as a capping agent and AA as a reducing agent. A calculated amount of seed particles were added to the solution containing metals precursors, H_2_PdCl_4_ and CuCl_2_, with AA and CTAB for the growth of the alloy. CTAB acts not only as a capping agent but also slows the reduction process of the Pd precursor through its coordination with Pd (II) and forms a [CTA]_2_PdBr_4−x_Cl_x_ complex. Diverse bimetallic Pd-Cu nanostructures are synthesized by altering the Pd and Cu precursor ratio as well as the pH of the reaction. The concentration of copper was kept constant whereas the concentration of the Pd precursor was increased and the ratio of Pd: Cu maintained from 1:10 to 3:2. There was a gradual increase in the size due to enough availability of the Pd precursor for deposition.

Reaction pH was monitored by varying HCl concentrations in the reaction. When the reaction pH was less (<2), polyhedral structure was favored, while a gradual increase in the reaction pH (2–3) saw the formation of multi-branched and octopodal structures. This happened because at a lower pH, the slow reduction rate allows the metal atoms to diffuse to a lower energy site throughout the growth, forming a polyhedral structure. The cubic Pd remained at the center and acted as seed for the growth of Cu. The metal with a higher concentration remained in the core part of the structure and the metals with less concentration shifted to the tips of the nanostructures as a shell.

#### Nanorods

Au-Pd bimetallic nanorods were prepared by the co-reduction of Au and Pd using the seed-mediated method where the growth was restricted kinetically (Sun et al., 2017). First, single crystalline seeds of Au nanorods were synthesized by mixing 0.5 mM HAuCl_4_ with 0.2 M CTAB in (1:1) volume ratio. Then, freshly prepared 0.6 mL (0.01 M) NaBH_4_ in water was injected into the prepared Au (III)-CTAB mixture with continuous stirring at 12,000 rpm. The color of solution altered from yellow to brownish yellow. Then, the seed solution was allowed to grow for 30 min (Ye et al., 2013). After the synthesis of single crystalline seeds of Au nanorods, multifaceted Au-Pd bimetallic alloy shells were overgrown via seed mediated electroless plating. The electroless plating was conducted by mixing metal precursors, HAuCl_4_ and H_2_PdCl_4_, with CTAB and AA at 30°C under ambient air. Since Au nanorods hold diverse facets, including local high-index and low-index facets, these can be tuned with different parameters to engineer into diverse architect with the overgrowth of the second metal in the growth step. Metal precursors ratio, reducing agent concentration, and the capping surfactants are responsible for the thickness dependent growth and surface co-deposition. These parameters also influenced atomic coordination and the stoichiometry maintained in the compositional form, resulting in different polyhedral nanostructures. For example, varying CTAB concentrations alters packing densities on different facets which favors growth in different geometries in BNS. CTAB forms bilayers on the surface of Au nanorods and regulates the diffusion rate and co-deposition ensuing alloy of Au-Pd. Surface growth of metals on Au nanorods leads to the overgrowth of nanocrystals and transforms into structurally distinct multifaceted geometries of Au and Pd alloy and core-shell nanorods. The electroless deposition of Au-Pd alloy over the Au nanorod seed was manipulated kinetically, which possibly resulted in huge families of polyhedral Au-Pd bimetallic nanorods with fine geometries including elongated hexoctahedral, truncated concave cuboidal, and truncated cuboidal nanorods.

#### Nanorings

Nanocrystals with hollow interiors have attracted great interest as they contain a larger surface-to-volume ratio in comparison to the solid counterpart which crucially affects the performance in different applications (Banaee and Crozier, 2010). Au nanorings are active materials and show significant tunability in localized surface plasmon resonance (LSPR) properties connected to variable transverse and longitudinal axes (Ozel et al., 2015). Wang et al. studied a seed-mediated route for the synthesis of Pd nanosheets supported by Au nanorings (Wang W. et al., 2016). The island growth of Au NPs due to the selective deposition on the sidelines of Pd nanosheets formed Pd ultrathin nanosheet supported by Au nanorings. Pure Au rings were further synthesized by selectively removing Pd cores by etching the Pd with excess nitric acid (HNO_3_) at room temperature. The expansion of Au rings over the Pd nanosheets was monitored in the ultraviolet-visible (UV-Vis) spectroscopy. The nanosheets formed from Pd showed a large LSPR band around ~940 nm. As the Au NPs slowly grown on the Pd nanosheets surface were covered completely, Pd peak disappeared due to the plasmon interaction between the Au and Pd nanosheets and two distinct peaks appeared at 520 and 770–915 nm. The peak appeared at 520 nm was due to the out of plane dipole mode of the Au rings and peak in the near infrared region was due to the combination of the in-plane dipole and face resonance modes in the nanorings (Ozel et al., 2015).

#### Nanobipyramids

**Figure 3:**
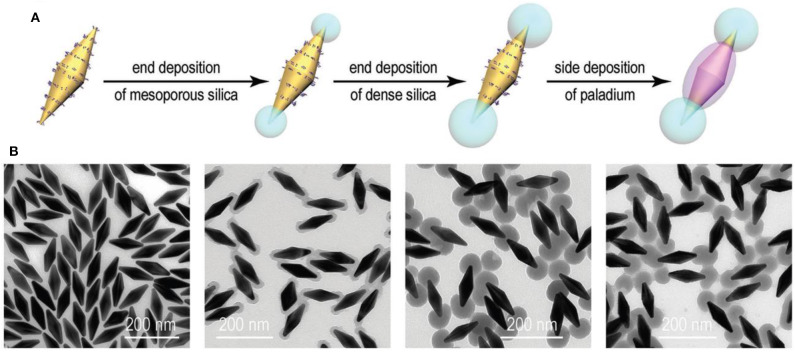
Side Pd deposition on the Au NBP/end-SiO_2_ nanostructures. **(A)** Schematic illustration of the process for side Pd deposition. **(B)** TEM images of the Au NBP, Au NBP/end-mSiO_2_, Au NBP/end-dSiO_2_, and Au NBP/side-Pd nanostructure samples [Reprinted with permission from Zhu et al. (2017), Copyright 2010, Advanced Functional Materials].

Anisotropic Au/SiO_2_/Pd nanobipyramids (NBPs) were successfully synthesized through the seed-mediated growth mechanism with a silica coating, which acts as a hard template to provide a preferential deposition of Pd over exposed surfaces of Au NBPs. The first step was the formation of Au NBPs seed by the reduction of a gold (HAuCl_4_) precursor with ice cold NaBH_4_ in the presence of trisodium citrate. Then, the seed solution was injected into the growth solution containing CTAB, HAuCl_4_, AgNO_3_, HCl, and AA (Zhu et al., 2017). Synthesis of Au NBP/mesoporous silica (mSiO_2_) involved coating of the silica [through tetraethyl orthosilicate (TEOS)] in sideline of Au NBPs surface with the help of effective blocking of methoxy poly(ethylene glycol) (mPEG-SH) to the ends. mPEG-SH has a tendency to bind preferentially to the ends of Au NBPs, which is very important in further side growth of silica on the surface of Au NBPs. The synthesized anisotropic Au NBP/mSiO_2_ further used as a hard template for position selective deposition of Pd on Au NBPs. The schematic representation for the synthesis of Au NBP/side-Pd nanostructures is shown in Figures 3A,B shows TEM image of the Au NBP, Au NBP/end-mSiO_2_, Au NBP/end-dSiO_2_, and Au NBP/side-Pd nanostructure. In addition to the small amount of TEOS on mSiO_2_ components, there was sequential deposition of dense SiO_2_ at the end. Finally, succeeding Pd deposition was directed by the dense silica components that occurred on the side surface of the Au NBPs.

#### Janus Star-Sphere Nanoparticles

Janus nanostructures that possess two or more different surface areas containing different optical and electronic properties are interesting candidates in diverse technological and biomedical applications. Janus nanoparticles with an iron oxide nanosphere and a branched gold nanostar (referred as Janus magnetic nanostar) was successfully synthesized by the two step seed-mediated-growth method (Reguera et al., 2017). Gold nanospheres were synthesized first and used as seeds for the growth of gold-iron oxide nanodumbbells. Subsequently, it acted as seeds for the directional growth of asymmetric gold nanostars: (1) The synthesis of asymmetric nano dumbbells involves a reaction between 1-octadecane, oleylamine, 1,2-hexadecanediol, oleic acid, and the two metal precursor Fe(CO)_5_, HAuCl_4_ at 300°C under N_2_ atmosphere. (2) The Janus magnetic nanostars synthesized by injecting nano dumbbells solution redispersed in chloroform with carboxyl terminated polyethylene glycol (PEG) into *N, N*-dimethylformamide (DMF) gold solution containing PVP after 1h of stirring. The addition of carboxyl-terminated-PEG to the nanodumbbells provides a good dispersion in DMF that evades the obscurity in phase-transformation of nanodumbbells, as these are stabilized by oleic acid and oleylamine in water.

### Galvanic Synthesis Method

Among the diverse methods available to achieve highly ordered anisotropic nanostructures, galvanic replacement reactions (GRR) has emerged a powerful method for molecular engineering which provides diverse opportunity for fabrication of anisotropic BNS with porous walls and hollow interiors. The term galvanic was derived from an Italian physician, Luigi Galvani, a biologist, philosopher, and pioneer of bioelectricity who established the first galvanic cell in the year 1780 (Focaccia and Simili, 2007). The GRR consists of a redox process between two metals where the lesser noble metal has the tendency to reduce the more noble metal cation having a higher redox potential with a suitable driving force (Liu and Astruc, 2017). The driving force here developed from the reduction potential difference between the two different metals where one acts as a sacrificial template and the other is metal cation (Da Silva et al., 2017). The sacrificed metal is corroded when it interacts with the ions of the second metal in the solution phase. This effortless reaction path can be used for the selection of metal ions to synthesize diverse anisotropic BNS. Manipulating the precursor feed ratio, the final product composition and morphology can be tuned. The shape of the nanostructures can be customized by providing a sacrificial template of distinct geometries (Da Silva et al., 2017). The template allows for restrictive oxidation and dissolution of atoms from particular sites because these particular sites have different chemical reactivities. This difference arises due to the shielding of the template by the deposition of the second metal during GRR, forming a layer at a specific location. Consequently, deposition of the metal atom on the template forms a porous shell with the same morphology, resembling its parent template with a slight rise in the dimensions.

When a metal cation interacts with a metal that has a lower reduction potential with respect to itself, it prefers to reduce first via the standard GRR route and accumulate in the core (Sun and Xia, 2002, 2004). For example, GRR between Ag NPs and ${\text{AuCl}}_{4}^{-}$ ions, two half equations occur as follows (Lu et al., 2007; Da Silva et al., 2017).

(1) $${\text{Ag}}_{\left(\text{aq}\right)}^{\text{+}}+{\text{1e}}^{-}\to {\text{Ag}}^{\text{0}}\;\varepsilon_{\text{1}}^{\text{0}}\text{=0}.\text{8V}$$

(2) $${\text{AuCl}}_{{\text{4}}^{-}}{}_{\left(\text{aq}\right)}+{\text{3e}}^{-}\;\to {\text{Au}}^{\text{0}}+{\text{4Cl}}_{\left(\text{aq}\right)}^{-}\;\varepsilon_{\text{1}}^{\text{0}}\text{=1}.\text{0V}$$

Resulting the final balanced equation:

(3) $${\text{AuCl}}_{{\text{4}}^{-}}{}_{\left(\text{aq}\right)}+{\text{3Ag}}^{\text{0}}\to \;{\text{3Ag}}_{\left(\text{aq}\right)}^{\text{+}}+{\text{Au}}^{\text{0}}+{\text{4Cl}}_{\left(\text{aq}\right)}^{-}\;\varepsilon_{\text{1}}^{\text{0}}\text{=0}.\text{2V}$$

Ag NPs in contact with ${\text{AuCl}}_{4}^{-}$ in solution, the Ag^0^ oxidized to ${\text{Ag}}_{\left(\text{aq}\right)}^{+}$ ions and ${\text{AuCl}}_{4}^{-}$ reduced to Au^0^ as shown in reaction (3), due to GRR driven by the electrochemical potential difference facilitating in the formation of cavities. Reduced Au^0^ atoms accumulated on the surface of Ag NP. As both Ag and Au have a face centered cubic (fcc) crystal structure and very close lattices constants (a_gold_ = 4.079 Å; a_silver_ = 4.086 Å), they easily form an Ag-Au alloy (Sun and Xia, 2004). A wide variety of nanostructures of Au, Pd, and Pt with unlike properties have been synthesized using different geometries of Ag as the template (Prevo et al., 2008; Zhang W. et al., 2012; Xia et al., 2013). A novel cellular like gold nanofeet was synthesized by a galvanic replacement reaction between Au@Ag core-shell and Au^+^. The Ag from the surface reduces the Au^+^ resulting in further growth of the Au shells on the Au/Ag core shell nanorods (Zheng G. et al., 2012). GRR, combining with co-reduction and Kirkendall processes, are used to synthesize wide varieties of nanostructures of different surface morphologies (González et al., 2011; Liu et al., 2013; Nafria et al., 2016). A few examples discussed below provide a better understanding to the classic GRR mechanism.

#### Nanosphere

**Figure 4:**
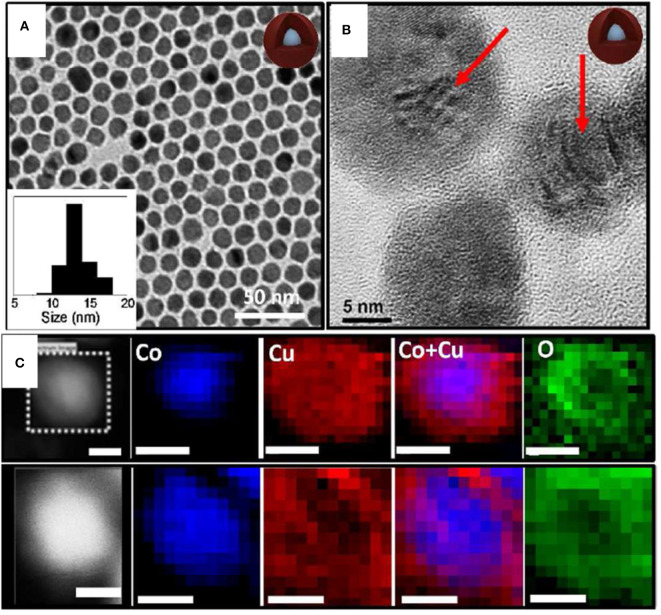
**(A)** TEM micrographs and size histograms (insets) of the Co_0.6_-Cu_0.4_ NPs. **(B)** HRTEM micrograph of Co-Cu NPs. **(C)** Two examples of HAADF (Z contrast) images and Co, Cu, and O electron energy loss spectroscopy (EELS) compositional maps of Co_0.6_-Cu_0.4_ NPs. Scale bars correspond to 5 nm [Reprinted with permission from Nafria et al. (2016), Copyright 2016, Langmuir].

Monodispersed Co-Cu NPs similar to a spherical shape with controlled metal ratios using a facile GRR technique were studied (Nafria et al., 2016). At first, Co NPs were synthesized using trioctylphosphine oxide (TOPO), oleylamine (OLA), and 1,2 anhydrous dichlorobenze (DCB) and were degassed in an air-free vacuum environment. Further, the solution was heated up to 180°C with rapid injection of Co_2_(CO)_8_ dissolved in DCB. The synthesis of the bimetallic involved injection of different stock solutions of Cu^+^ ions in OLA to Co spherical NPs and was heated up to 180°C. Upon the addition of Cu^+^ to Co NPs, Co oxidized to Co^2+^and a Cu shell was grown due to GRR. The driving force for the substitution of Co by Cu^+^ ions was due to the superior reduction potential of Cu^+^ than Co^2+^ (at 25 °C and 1 atm, *E*° (Co^2+^/Co) = −0.28 V; *E*° (Cu^+^/Cu) = 0.52 V). TEM images of the bimetallic Co-Cu NPs is shown in Figure 4. The average size of Co NPs was 10 nm and, with the addition of ${\text{Cu}}_{,}^{+}$ the size slightly increased from Co NPs as shown in Figure 4A. HRTEM image (Figure 4B) confirmed the presence of Moire fringes in Co-Cu NPs, concluding that two crystalline structures are confined together. The planes of (011) and (111) with d-spacing of 2.44 and 2.98 Å matches with Cu_2_O shell. The elemental mapping of bimetallic Co-Cu NPs in Figure 4C shows Co was confined in the core whereas partially oxidized Cu resided in the shell.

#### Nanoboxes

Porous hollow structures are very important due to two important features. One is the open hollow interior that allows access inside of the nanostructures and second is the distribution of metal atoms different from bulk nanostructures. The known fact is that porosity in the material develops due to differential solid-state diffusion rates of the reactants involved during the oxidation or alloying reaction. Due to this difference, it develops an interfacial motion between the two participating metals, which is known as Kirkendall effect. This effect was reported by Smigelkas and Kirkendall in the year 1947 for copper and zinc in brass, due to the difference in the diffusion rates of these two metals resulting in the movement of the interface (Paul, 2004). The atomic diffusion is accompanied by vacancy exchange and not by the interchange of atoms. When there is flow of matter in a particular direction, it is balanced by the reverse flow of vacancies which reduces into the formation of pores. Edgar González and co combined the mechanism of galvanic exchange and Kirkendall growth technique to form very unique structures of PdAuAg nanoboxes of a double-walled morphology (González et al., 2011). In this protocol, Ag was used as a template and Au, Pd, and Pt served as oxidizing agents in order to synthesize hollow nanostructures. The synthesis of trimetallic nanoboxes involved three steps: (a) Mixture of CTAB and AA added into aqueous solution containing silver metal precursor; (b) Addition of second metal precursor, HAuCl_4_ with a flow rate from 20 to 50 μL/min; and (c) Addition of Pd salt with a flow rate from 200 μL/min to 270 μL/min and followed by isolation of synthesized nanostructures.

**Figure 5:**
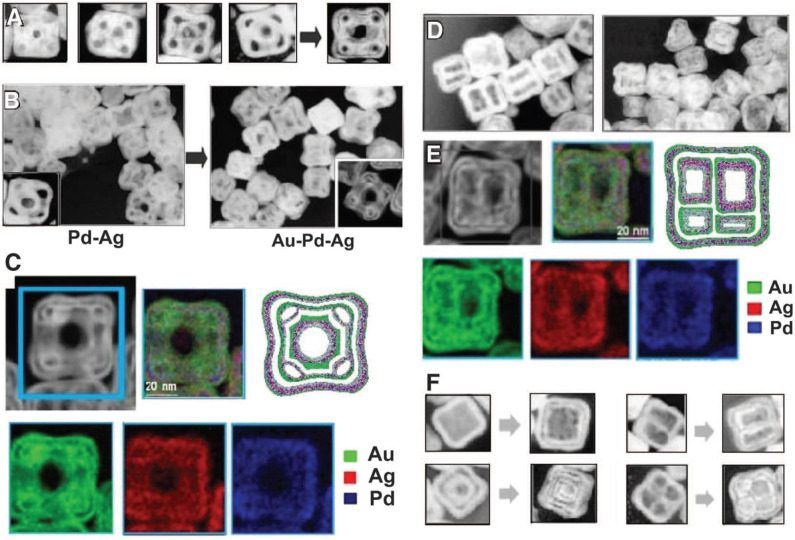
**(A)** TEM images illustrating the stages of formation of hollow nanostructures of Au-Ag-Pd nanoboxes by sequential action of galvanic replacement and Kirkendall effect. **(B)** TEM images showing the nanostructures dominated by galvanic replacement using Pd (left) and by Kirkendall effect using Au (right). **(C)** HAADF-STEM image and corresponding EDX elemental maps. **(D)** TEM images of multichambered NPs. **(E)** HAADF-STEM detail and corresponding EDX elemental maps. **(F)** TEM images of different structures produced by sequential action of galvanic replacement and Kirkendall effect [Reprinted with permission from González et al. (2011), Copyright 2011, Science].

Initially oxidation of Ag was favored since it has less positive reduction potential. Ag was oxidized and Au^3+^ was reduced since the electron transferred from the less noble metal Ag and was accepted by Au^3+^, as both Au and Ag have a fcc crystal structure and very close lattice constant value. Au^0^ deposits over Ag, forming a fine thin layer. Formation of the thin layer over Ag restricts further oxidation of Ag. As the area of the electrode increases, the corrosion becomes stronger in the part of Ag which is exposed and uncovered by Au^0^, resulting in the formation of pinholes forming void in Ag cubes. Thus, a galvanic replacement takes place between Ag and Au. The released electron migrates to the surface of the nanocube and is attracted by ${\text{AuCl}}_{2}^{-}$, resulting in Au^0^ growth further on the Ag template due to preferential nucleation. After the formation of cavities via GRR and the successive formation of Au coating, growth of a thin film by Ag starts due to diffusion intermetallic coupling resulting in the Kirkendall cavity. Trimetallic structures of Pd-Au-Ag synthesized with the same mechanism by the addition of both Au and Pd precursors simultaneously or sequentially as shown in Figure 5.

#### Nanotubes

**Figure 6:**
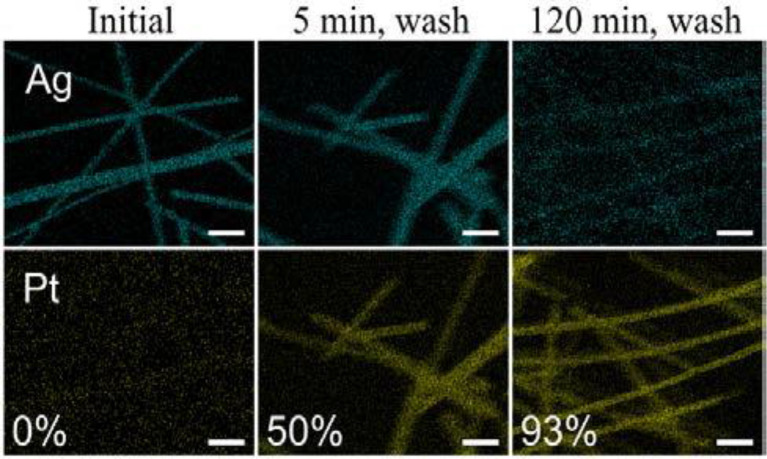
Elemental mapping from SEM of PtAg nanotubes made via SGD of Ag nanowires after reaction times of 0, 5, and 120 min, with Pt wt% listed. The 5- and 120-min samples were washed with a sodium chloride (NaCl) solution then refluxed in HNO_3_ to remove residual Ag on the surfaces of the nanotubes. Scalebars are 500 nm [Reprinted with permission from Strand et al. (2016), Copyright 2016, The Journal of Physical Chemistry].

The synthesis of PtAg nanotubes occurred via the spontaneous galvanic displacement (SGD) method, where Ag nanowires (NWs) were displaced by Pt (Strand et al., 2016). The report explains the mechanism for the formation of Ag nanowires using K_2_PtCl_4_. The growth of an uneven surface over the nanotubes was facilitated by the replacement of Ag by Pt during SGD reaction. In the synthesis, commercially available Ag NWs were used as a displacement template during the SGD reaction by Pt. Initially, Ag NWs were dispersed in the DI water (1:1 w/V ratio) and heated to 90°C in the oil bath. Then the solution was injected with an aqueous solution of K_2_PtCl_4_ and the reaction was carried out in different intervals of time (0, 2, 5, 10, 15, 60, and 120 minutes) in order to determine the displacement mechanisms. Figure 6 shows the morphology of the nanotubes obtained from the SGD reaction and the elemental mapping revealed the greater part of displacement of Ag by Pt had occurred between 2 to 10 min with an increase in Pt wt% from time = 0 to 10 min.

### Antigalvanic Reduction Method

As discussed, GRR phenomenon is a spontaneous process, when atoms of one metal come into contact with another metal ion of a higher electrochemical potential in comparison to the metal atom in solution. The metal atoms oxidize and go into the solution phase and then the simultaneous reduction of metal ions are reduced to atoms and deposit over the metal template. Thus, diversity of template choice in the GRR method can result in an extensive range of nanostructures (Wu, 2012). Recently, a new phenomenon, antigalvanic reduction reaction (AGR), which contradicts to the galvanic principle, has been more appealing for the synthesis of BNS. Advanced research revealed that AGR is more relevant to mechanistic, synthetic, and catalytic purposes. The AGR mechanism deals with the metals ions which are lesser noble (more reactive) reduced by more noble metals (less reactive). This is completely opposite to the GRR and has not been explored widely. Murray and co-workers in the year 2010 revealed that an Au nanocluster [Au_25_(SC_2_H_4_Ph)_18_] reacts with Ag ions (Choi et al., 2010), follows the AGR mechanism. The well-known nanocluster Au_25_L_18_ i.e., [Au_25_(SC_2_H_4_Ph)_18_], Ligand L = SC_2_Ph (phenylethanethiolate) holds 13-atom icosahedral core surrounded by 6 Au_2_(SR)_3_ shell. When it reacts with Ag^+^, it forms Au_25_Ag(SC_2_H_4_)Ph)_18_, Au_24_Ag_1_(SC_2_H_4_)Ph)_18_, and Au_23_Ag_2_(SC_2_H_4_)Ph)_18_ with respect to an optimized ratio of Ag^+^/Au_25_${\text{L}}_{18}^{-}$. Au atoms reduced by Ag^+^ without any alteration of ligands confirms the AGR phenomena.

The first general theory of the AGR mechanism was predicted in 2012 by Wu et al., and stated that the thiolate ligands in [Au_25_(SC_2_H_4_Ph)_18_] might be responsible for supporting the AGR mechanism (Wu, 2012). The ligands attached on the surface of nanocrystals develop a partial negative charge to the Au and Ag nanocrystals. Wang and Wu suggested that in the AGR mechanism there is no need for reductive ligands as nanoparticles can reduce metal ions (Wang et al., 2014). Small NPs of more noble metals, such as Au and Pt, can reduce less noble metal ions like Ag^+^, but this is a deviation from the conventional free energy-based arguments used in galvanic displacement (Wu, 2012; Wang et al., 2014). Sun and coworkers studied a one pot approach using the AGR mechanism for an Ag-Au nanocluster (Sun et al., 2014), whereas Corpuz reported positively charged photoluminescent bimetallic Au-Ag nanoclusters synthesized based on the AGR mechanism (Corpuz et al., 2017). A core shell nanostructure of silver (shell) and gold (core), Ag@Au NPs synthesized and explained through AGR mechanism (Sahu and Prasad, 2015). This was carried out in order to determine whether the reduction phenomena of Ag^+^ is driven by ligand dodecylamine (DDA) or the surface atoms (Au^0^) of Au NPs acting as reducing agents in AGR reaction. The reaction was carried out using two different capping agents, dodecanethiol (DDT) and DDA. When DDT was used as a capping agent on Au NPs, there was no core shell formation. Instead individual Au and Ag nanoparticles resulted. But when DDA was capped on Au NPs, an Ag@Au core shell nanostructure formed. Hence, it is concluded that the reduction of Ag^+^ to Ag^0^ on the surface of Au^0^ was governed by DDA resulting in the final core-shell structure. The change in the ligand results in alterations in the nanostructures. Even if DDA has a stronger binding affinity with Au NP surface, during reflux condition DDA detaches from Au NPs leaving behind a naked Au NP exposed surface. Meanwhile, DDA does not possess binding affinity with an Ag^0^ surface which results in the generation of nascent uncapped Ag^0^ atoms in the solution. The nascent uncapped Ag^0^ atoms preferred to attach to the surface of naked Au NP resulting in an Ag@Au core shell nanostructure. On the other hand, when DDT was used as a capping agent, it possessed a strong affinity toward both Ag and Au nanoparticle surfaces. After the reduction of Ag^+^ ions to Ag^0^, DDT immediately caps Ag^0^ atoms in the solution, resulting in individual Au and Ag nanoparticles. The nature of ligand binding to the nanoparticle has a significant role in establishing the final structure.

### One Pot Synthesis Method

The synthesis of BNS involved two mechanisms: GRR and the co-reduction of two metal precursors in the presence of a reducing agent. However, GRR requires significant redox potential difference between two metals to drive the reaction which limits the feasible combinations of two metals and tunability in its elemental composition. Whereas, the second approach involves the tiresome cleaning process after synthesis. Thus, in order to improve the efficiency of any chemical reaction researchers tried one pot techniques in which reactants are subjected to successive chemical reactions in one reactor involving multiple steps. These methods have been mostly preferred due to the elimination of the prolonged separation procedure and purification of the additional compounds which can be done in less time and increases the yield of the reaction. Further, this method eases the reaction path in the preparation of BNS. However, it needs a harsh reaction environment for controlling the nucleation of metals that have different reducing behaviors. Using a one-step synthesis route, core-shell structures can be synthesized when the two metals have large differences in their standard redox potentials by providing a harsh reaction environment (He et al., 2010; Park et al., 2014).

#### Trigonal Bipyramid Nanoframes

**Figure 7:**
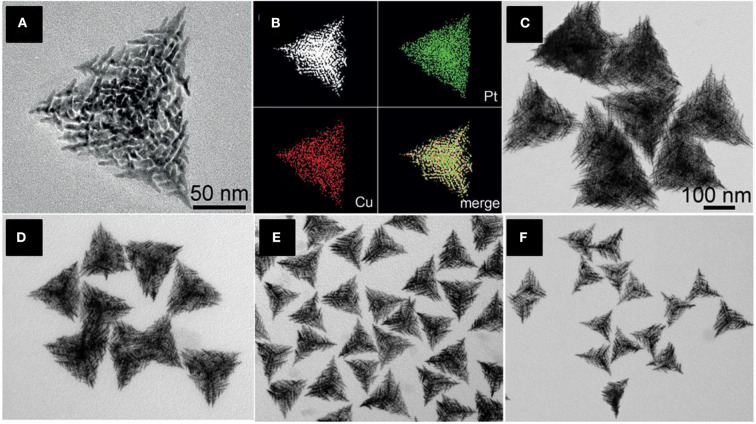
**(A)** TEM image of an individual Pt–Cu HTBNFs, **(B)** The corresponding HAADF-STEM image and elemental mapping showing the distribution of Pt (green) and Cu (red). **(C–F)** TEM images of Pt-Cu HTBNFs obtained through the addition of different amounts of KI aqueous solution in the synthesis while keeping the other parameters constant: **(C)** 30 mL-250 nm, **(D)** 50 mL-200 nm, **(E)** 100 mL-150 nm, and **(F)** 150 mL-110 nm. The scale bar in **(C)** also applies to **(D–F)** [Reprinted with permission from Chen et al. (2015), Copyright 2015, Chemie International Edition].

Nanoframes have an ultrathin structure of large surface-to-volume ratios and high surface areas (Chen et al., 2015) and come in various sizes where the active atoms are more exposed over the surface. Hierarchical trigonal bipyramid nanoframes (HTBNFs) are formed using a Pt-Cu formed alloy using one pot synthesis method (Chen et al., 2015). The structure consists of a string of parallel structural units with sharp branch tips. CuCl_2_, H_2_PtCl_6_, and KI were added altogether with polyvinylpyrrolidone (PVP) and ethylene glycol and heated at 140°C for the formation of Pt-Cu HTBNFs. In this synthesis, the co-reduction of the precursor metals Pt and Cu lead the shape of alloy structure. Due to the large differences of the electrochemical reduction potential of ${\text{PtCl}}_{6}^{2-}$/Pt^0^ and Cu^2+/^Cu^0^, I^−^ forms a complex by coordinating with Pt^4+^ rather than Cl^−^, resulting in a closer reduction potential between [PtI_6_]^2−^/Pt^0^ and Cu^2+^/Cu^0^. The size of the HTBNFs was influenced by the amount of KI added in the synthesis. With the gradual addition of the KI solution, the size of the HTBNFs decreased from 250 to 110 nm as shown in Figure 7. Initially, miniature monopods, bipods, and tripods shaped structures were formed, and the development of secondary, tertiary, and higher-organized branches were formed confirming the final HTBNF structures. Metal precursors (H_2_PtCl_6_ and CuCl_2_) ratio played a vital role in the development of different Pt-Cu HTBNFs.

#### Nano Dendritic Core-Shell

Various protocols have been used for the synthesis of Pt-based bimetallic core-shell nanospheres using Pt as a shell. Core-shell nanostructures where Pt develops as a dendritic shell have been studied. A Pt based bimetallic core-shell required an elevated temperature and pressure to optimize the synthesis protocol. Catalytic activity of the Pt-based core shell depends on the thickness of the shell. The thinner the Pt shell, the better the catalytic activity. Using the one pot inexpensive synthesis method, Au-Pt nanocolloids with Au as core and Pt as a dendritic shell was synthesized (Ataee-Esfahani et al., 2010). Au-Pt nanocolloids formed by mixing chloroplatinic acid hexahydrate (H_2_PtCl_6_.6H_2_O) and HAuCl_4_ precursors and growth was controlled by the low concentration of surfactant with an ultrasonic irradiation. Altering the Pt and Au molar ratios in the reaction, the shell thickness has been controlled. AA and pluronic F127 were used as a reducing agent and surfactant/structure directing agent, respectively. The formation of the core-shell is driven by the reduction potentials difference of the two dissimilar metal precursors, H_2_PtCl_6_ and HAuCl_4_. The reduction of ${\text{AuCl}}_{4}^{-}$ and [PtCl_6_]^2−^ can be explained by the subsequent reactions as follows (Ataee-Esfahani et al., 2010).

(4) $${\text{AuCl}}_{4}^{-}+{\text{3e}}^{-}\to \text{Au}+{\text{4Cl}}^{-}\;+\text{1}:\text{00eV vs}.\text{ SHE}$$

(5) $${\left[{\text{PtCl}}_{6}\right]}^{\text{2}-}+{\text{2e}}^{-}\to {\left[{\text{PtCl}}_{4}\right]}^{\text{2}-}\;+{\text{2Cl}}^{-}+\text{0}.\text{68eV vs}.\text{ SHE}$$

(6) $${\left[{\text{PtCl}}_{4}\right]}^{\text{2}-}+{\text{2e}}^{-}\to \text{Pt}+{\text{4Cl}}^{-}\;+\text{0}.\text{76eV vs}.\text{ SHE}$$

Due to the large reduction potential difference between Au and Pt, Au cations reduced faster to form Au core, while Pt cations reduced later and formed overgrowth as a dendrite structure on the surface of the Au core. Pluronic F127 acts as a structural-directing agent, resulting in crown-like ethers conformation (Wang et al., 2010).

**Figure 8:**
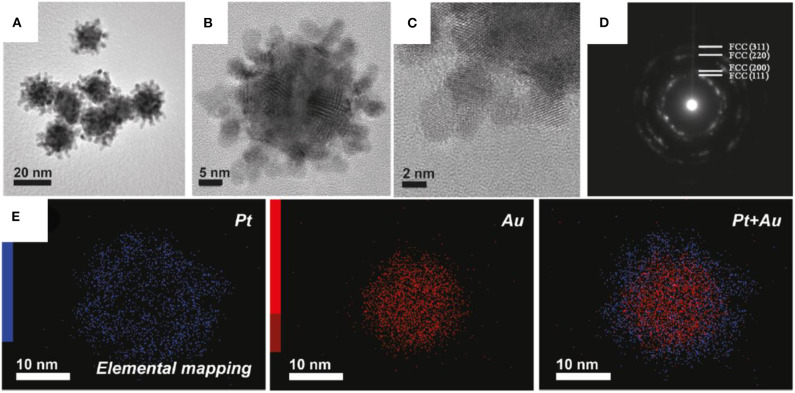
**(A)** Low-magnification and **(B)** high-magnification TEM images of Au-Pt nanocolloids, **(C)** High-magnification TEM image of the Pt shell, **(D)** Selected-area electron diffraction (SAED) patterns taken from the Pt shell region, **(E)** elemental mapping of Au-Pt nanocolloids, the red color indicates Au, whereas the blue color indicates Pt [Reprinted with permission from Ataee-Esfahani et al. (2010), Copyright 2010, Chemistry of Materials].

The interaction linking hydrophobic polypropylene oxide (PPO) groups and the Pt surface is through adsorption on the Pt surface. Due to which cavities are formed following the formation of Pt dendrites, such phenomena is restricted in the case of Au as F127 cannot act as a capping agent due to Au core forming an irregular nanostructure. Figures 8A,B shows the TEM image of Au-Pt nanocolloids. The size of Au-Pt nanocolloids are in the range of 20–35 nm. HRTEM images in Figure 8C shows the lattice fringes of an individual Pt shell corresponds to (111) plane with face-centered cubic (fcc) crystal. A ring like pattern was observed in SAED pattern (Figure 8D) indicating the different planes such as (111), (200), (220), and (311) of Pt confirms the polycrystalline nature. Elemental mapping in the Figure 8E shows Au is present at the center as a core and Pt forms dendritic shell leading to an Au-Pt core-shell.

#### Nano Dendritic Alloy

**Figure 9:**
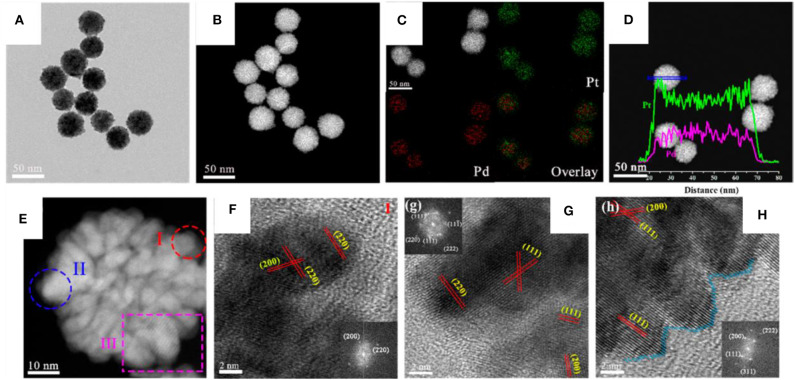
**(A)** Bright-field TEM image and **(B)** HAADF-STEM image of Pt_3_Pd_1_nanodendrites, **(C)** HAADF-STEM image and EDX elements mapping and **(D)** line-scanning profiles. **(E)** The individual Pt_3_Pd_1_nanodendrites and **(F–H)** the corresponding local HR-TEM image. The insets in **(F–H)** show the corresponding FFT pattern [Reprinted with permission from Wu et al. (2019a), Copyright 2019, ACS Sustainable Chemistry and Engineering].

Pt-Pd bimetallic nanodendrites were synthesized using the one-pot solvothermal method as shown in Figure 9 (Wu et al., 2019a). Three different nanoporous Pt-Pd bimetallic nanodendrite structures were obtained by varying the precursor ratio of Pt and Pd in an ethylene glycol solution in the presence of iodide ions and polyvinylpyrrolidone (PVP). When the ratio between Pt/Pd was maintained to 3:1 in the standard reaction, uniform porous spherical nanodendrites with particle sizes of 44.9 ± 2.6 nm were formed. Further, changing the Pt/Pd ratio to 2:1 and 1:1, the shape and size varied to nanodendrites with coarser branches of particle size of 34.3 ± 2.1 nm and nanodendrites with quasi-cubic shape branches of particle size of 25.1 ± 1.8 nm, respectively. Here, Iodide ion has shown its vital role in the formation of dendrite structure. Absence of iodide ion results in NPs with uniform polyhedral nanostructures of particle size 4.2 ± 1.8 nm. But in addition to I^−^ to the reaction, Pt^4+^ and Pd^2+^ ions form complexes, [PtI_6_]^2−^ and [PdI_4_]^2−^. Iodide ions further alter the reduction sequence and decrease the reduction rate of metal precursors and formed Pt-Pd bimetallic nanodendrites. Dendritic alloy was reported by Kamel Eid and coworker in 2016 using the one pot co-reduction technique. The synthesized porous dendritic bimetallic Pt-Ni nanocrystals are highly efficient catalysts in oxygen reduction reaction (ORR) (Eid et al., 2016). The aqueous solution of K_2_PtCl_4_, Ni(NO_3_)_2_, PVP, and AA under ultrasonic irradiation or magnetic stirring created dendritic or flower like nanocrystals of Pt-Ni synthesized, respectively. The synthesized structures were reported to show a very good electrocatalytic activity in ORR.

#### Core-Shell Nanocubes

Bimetallic nanocubes with high-surface energy facets were difficult to synthesize unless the two metal ions were co-reduced in precisely under-controlled kinetic environments. The nucleation of metal and controlling of the kinetic growth in the presence of multiple metal precursors is the great challenge. Park and coworkers employed the one pot protocol in an aqueous room temperature solution and synthesized Au-Pd core-shell nanocubes (NCs) with a truncated hexoctahedral (THOH) structure (Park et al., 2014). For the synthesis of THOH Au@Pd NCs, a mixture of KAuBr_4_.xH_2_O, K_2_PdBr_4_, and CTAB in an aqueous solution was treated with AA and NaOH (molar ratio 4:1) to reduce two precursor metal ions. The solution was shaken for a few seconds and kept undisturbed for 1 h. The synthesized solution was centrifuged to remove excess unreacted ions. The manipulation of the molar ratio among the two metals, reducing agents, and capping agent provided a control over the nucleation and the kinetic growth resulting in the formation of THOH Au@Pd NCs. The morphology restricted to the core shell because the Au precursor possesses higher reduction kinetics compared to the Pd precursor in the provided experimental condition. The Au precursor preferentially reduces first to form an Au NC core confined to hexoctahedral (HOH) which are employed as seeds for further growth. Pd has an essential role in establishing high-index facets during the growth. In the absence of the Pd precursor, irregular morphology of Au NCs formed. This happens because Pd selectively grows on the sites of high energy facets of Au NCs resulting in an increase in the stabilization of high-index facets. As a result of this, the crystal structure kinetically entraps in the HOH structure (Lee et al., 2013).

#### Nanoboxes

**Figure 10:**
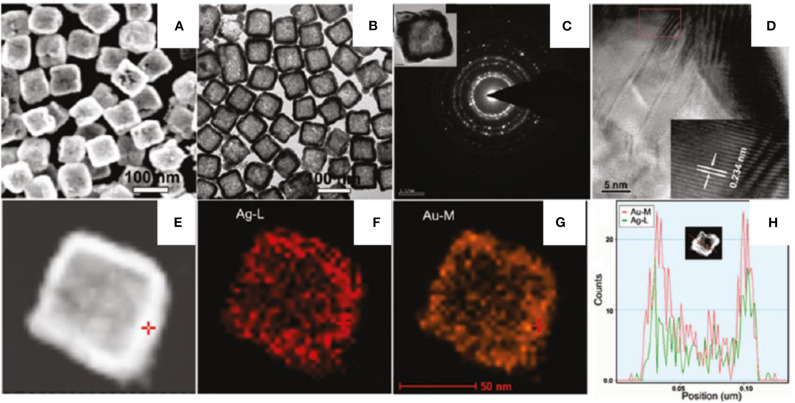
**(A)** SEM and **(B)** TEM images of AgAu nanoboxes; **(C)** ED pattern of a single nanobox; **(D)** HRTEM image near the box wall; **(E)** STEM-HAADF image; **(F,G)** STEM-EDX element mappings of Ag and Au, respectively; and **(H)** cross-section composition line profiles of one nanobox [Reprinted with permission from He et al. (2010), Chemistry of Materials].

The one pot facile synthesis technique was employed to synthesize AgAu nanoboxes (He et al., 2010). The synthesis proceeds with a mixture of two metal precursors, HAuCl_4_ and AgNO_3_ (molar ratio 1:1), in an aqueous solution and AA is immediately added under vigorous shaking. The appearance of a blue color confirms the formation of AgAu NPs stabilized by CTAB (0.1 M). Ag/Au molar ratio is the key parameter to control the shape of AgAu NPs. Star-like solid NPs formed at a lower Ag/Au molar ratio of 0.2 and increased the molar ratio of Ag/Au from 0.4 to 0.8, resulting in nanoboxes forming. Uniform nanoboxes are formed at an Ag/Au ratio of 1/1 (Figure 10). It confirms that the involvement of Ag is highly important in the formation AgAu nanoboxes.

Figures 10A,B shows the SEM and TEM images of AgAu NPs confined to a cubic morphology with holes over the surface in which the edge of the nanoboxes is more highly dense than the center. The regular size of nanoboxes is 75 ± 5 nm with a shell thickness of 9.6 ± 2.1 nm. The electron diffraction (ED) pattern confirms (Figure 10C) the polycrystalline nature of the nanoboxes. The d spacing of lattice fringes near walls (Figure 10D) is 0.234 which corresponds to (111) plane of Au or (111) plane of Ag (0.233 nm).

### One Pot Hydrothermal Technique

The hydrothermal technique is the most important tool for the synthesis of anisotropic nanostructures (Byrappa and Adschiri, 2007). The hydrothermal technique not only helps in processing monodispersed and highly homogeneous nanostructures but also provides a reaction condition in order to acquire hybrid and nano-amalgamated materials. The technique involves a heterogeneous reaction and is subjected to high pressure and temperature in aqueous solvents which dissolve and recover the materials. In hydrothermal synthesis, all the reactants are dissolved in water or any other suitable solvent and sealed in a vessel for the synthesis of the desired shape, size, and structure (Byrappa and Adschiri, 2007). Products of low impurity, monodispersity, smaller particle size, high activity, and fewer defects with simple purification shows the advantages of the hydrothermal method over other synthetic approaches. Synthesis of Pt-Pd concave nanocubes (Wu et al., 2019b), Au-Pd alloy and core-shell (Kuai et al., 2012), and Pd-Rh nanoframes and cubes (Ye et al., 2015) have been synthesized through the hydrothermal method as discussed below.

#### Concave Nanocubes

Pt and Pd can be effortlessly shaped into a bimetallic alloy structure and both are known for their catalytic behavior. The catalytic performance to a large extent depends on the shape, size, structure, and morphology of the bimetallic structure. A facile one pot method is used for Pt-Pd symmetry broken concave nanocubes (SBCNCs) synthesis (Wu et al., 2019b). A solution of disodium tetrachloropalladate (Na_2_PdCl_4_), H_2_PtCl_6_.6H_2_O, and sodium iodide (NaI) in N-dimethylformamide (DMF) was used for the hydrothermal reaction at 130°C. Pd (II) cannot be reduced before Pt (IV) due to their respective standard reduction potentials (E^0^) between Pd^2+^/Pd and Pt^4+^/Pt. As iodide ion and Pt (IV) forms a complex in an aqueous medium, favored by aqua and hydroxo substitution (Huang et al., 2009; Straney et al., 2014; Zhang J. et al., 2016), and hence, it is easier to reduce Pd before Pt. Capping agent I^−^ prefers to grow along (100) direction forming Pt-Pd nanocubes in the beginning. But an unstirred solution provides a slow surface diffusion rate which escorts the directional growth toward the vertexes and stacks atoms to the corner of the nanocubes, leading to the arrangement of asymmetric concave nanocubes.

#### Alloy and Core-Shell

To Minimize the two-step epitaxial growth mechanism which has the limitation of large-scale synthesis, Long Kuai and co approached the one pot hydrothermal co-reduction technique for the synthesis of Au-Pd alloy and core-shell nanostructures (Kuai et al., 2012).

**Figure 11:**
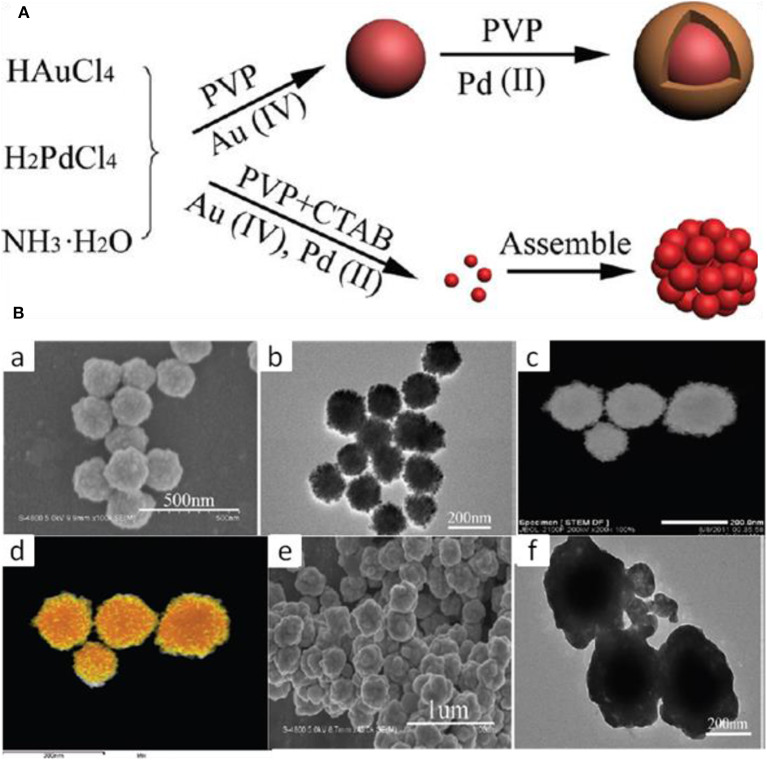
**(A)** The formation Process of Au–Pd Core–Shell and Alloy BNS, **(B)** Morphology analysis, (a) SEM, (b) TEM, (c) HAADF-STEM, and (d) HAADFSTEM-EDS mapping images of Au–Pd alloy nanostructures. (e) SEM and (f) TEM images of Au-Pd core-shell nanostructures [Reprinted with permission from Kuai et al. (2012), Copyright 2012, Langmuir].

HAuCl_4_ and H_2_PdCl_4_ were used as the metal precursors and PVP as the reductant for the synthesis of core-shell nanostructures. The addition of CTAB to the reaction containing HAuCl_4_, H_2_PdCl_4_, and PVP alloy structure formed instead of core-shell. The size of the core-shell was larger than the alloy, as shown in Figure 11. Hence, the reaction proceeds core-shell formation naturally, but the addition of CTAB restricts the formation of core-shell and favors the formation of alloy. This is due to the stronger reducing activity of CTAB than PVP under hydrothermal conditions and both the metals reduced together, forming alloy nanostructures.

#### Nanoframes and Nanoboxes

Hollow metallic nanostructures are a significant research area due to their large surface areas with high reactivity (Sun and Xia, 2004). Both Pd and Rh act as significant catalysts and have been hardly studied in terms of bimetallic hollow nanostructures. Ye and coworkers reported the synthesis of Pd-Rh nanoframes and cubes using a hydrothermal reaction (Ye et al., 2015). Pd-Rh bimetallic nanoframes and nanoboxes were produced by selectively removing Pd cores from different Pd-Rh nanocubes. The average Pd-Rh nanocube size was 12.7 ± 1.3 nm. Further, selective elimination of Pd from the synthesized Pd-Rh nanocubes formed the cubic nanoframes. The size and shape of the nanoframes was intact from the parent nanocubes but the edges were fairly thin (i.e., 2 nm). During the synthesis, formaldehyde (HCHO) was used for altering the growth behavior and reaction kinetics to form different hollow nanostructures, nanoframes, or nanoboxes. The shape of Pd-Rh nanocubes was intact in the absence of HCHO. However, the size increased to 33.0 ± 3.8 nm due to the reduced nucleation from the weak reducing agent PVP. Rh-Rh has a strong interaction during etching and has more stability than Pd (Lu et al., 2013). As a result, Pd comes out preferentially from the nanocubes. However, Pd cannot be entirely removed from the nanocubes as Pd partly alloys with Rh throughout the synthesis of Pd-Rh nanocubes. After drawing Pd from Pd-Rh nanocubes, hollow structures were obtained (Xie et al., 2012).

#### Nanorods

Ni-Fe bimetallic phosphides (NiFeP) nanorods were synthesized at a low-temperature phosphorization process using a hydrothermal technique (Du et al., 2018). In the method, a Ni-Fe bimetallic-organic frameworks (MIL-88-Fe_2_Ni MOF) precursor was used to engineer NiFeP nanorod structures. The atoms of Ni and Fe were connected with the help of an organic ligand terephthalic acid. The procedure follows: (1) synthesis of the MIL-88-Fe_2_Ni nanorods precursor in which hydrothermal treatment of mixture FeCl_3_.6H_2_O, Ni(NO_3_)_2_.6H_2_O, 1,4-benzenedicarboxylic acid (H_2_bdc) in dimethyl formamide (DMF), and NaOH solution were carried out at 100 °C for 15 h. (2) The synthesized MIL-88-Fe_2_Ni nanorods was further calcined at 450 °C forming NiFe_2_O_4_ nanorods. (3) The final NiFeP was obtained by low temperature phosphorization of NiFe_2_O_4_ nanorods with NaH_2_PO_2_ as phosphorus source. The NiFeP nanorods showed outstanding electrocatalytic behavior during the hydrogen evolution reaction (HER) in basic medium. A synergistic effect developed from NiP_2_ and FeP was responsible for the excellent electrocatalytic performance. The Ni-Fe bimetallic phosphides (NiFeP) nanorods form an alloy of well distributed Fe, Ni, and P elements.

### Electrospinning Technique

The electrospinning technique is essentially used for fiber synthesis. Electric force is used to draw charged threads of material solutions or material melts up to fiber diameters in the order of a 100 nanometers using a polymer solution. This technique is widely recognized as a nano structuring technique because of its ability to direct the morphology and exterior topology to form fine fibers (Agarwal et al., 2009). There are two electrospinning arrangements: vertical and horizontal. Certain complex setups have been designed in order to get intricate nanofibrous structures with uniformity. The technique is maintained at room temperature under atmospheric conditions (Bhardwaj and Kundu, 2010). The system consists of three key apparatus: a high voltage power supplier, a spinneret, and a collecting plate which is grounded. It supplies a charge of a certain polarity to a polymer solution from a high voltage source. This is then collected by a collector of opposite polarity. The polymer fluid is supplied to the capillary tube for electrospinning and results in the formation of fiber. In order to achieve parallel bimetallic fibers, the whirling solution consists of two precursor salts with polymer compounds. Within polymer nanofibers, the reduction of salt produces NPs of diameters in the range of 3–5 nm. The dispersion of monometallic or bimetallic NPs in polymer nanofibers is of great interest for heterogeneous catalysis (Demir et al., 2004). In addition to the morphology, alteration of the elemental ratio in the mother solution provides wide tunability in the application using this technique.

#### Nanofibers

Carbon materials have attracted huge interest owing to their outstanding physicochemical properties studied as an electrocatalyst in fuel cells (Senthilkumar et al., 2018). Due to their high surface to volume ratio, carbon nanofibers have been widely used for extensive chemical characteristics (Ghouri et al., 2015).

**Figure 12:**
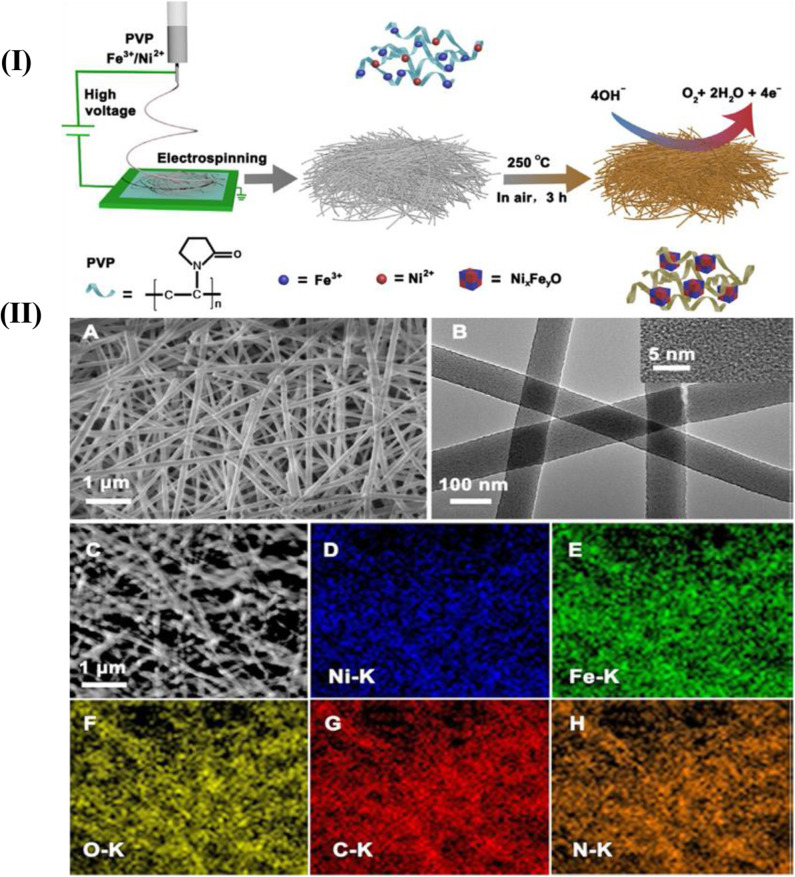
**(I)** Illustration of the synthesis of the Ni-Fe oxide/carbon composite nanofibers. **(II) (A)** SEM image and **(B)** TEM image and a high-resolution TEM image (inset) of Ni_1_Fe_2_-250. **(C)** Scanning transmission electron micrograph and **(D–H)** the corresponding elemental mapping images of Ni_1_Fe_2_-250 [Reprinted with permission from Chen et al. (2016), Copyright 2016, ChemCatChem].

Doping metal or bimetals with the carbon nanofibers enhances its properties. Pd-Cerium (Ce) bimetallic doped with carbon nanofibers synthesized using the electrospinning technique showed excellent electrochemical properties and high uniformity (Alvi and Akhtar, 2016). Cobalt (Co) has been recognized for its catalytic behavior from all other non-noble metal catalysts and Pd, being cheaper than Pt and also widely used as catalyst. Bimetallic nanofibers of Pd-doped Co nanofibers offered excellent photocatalytic behavior and were used as the active material for diode (Barakat et al., 2012). Hui reported bimetallic Ni-Fe oxide/carbon composite nanofibers with excellent OER catalytic behavior synthesized through the electrospinning technique. The advantage of this technique is the elemental proportions that can be precisely adjusted in the mother solution, which enhanced the OER activity (Chen et al., 2016). The molar ratio of Fe and Ni tuned to obtain a number of composite materials of different Ni/Fe atomic ratios for various structures. The whole process is demonstrated in Figures 12I,IIA-B shows the SEM and TEM image of synthesized Ni-Fe composite nanofibers calcined at 250°C and elemental mapping in Figures 12IIC–H shows the presence of Ni, Fe, O, C, and N in the composite material. The elements homogenously formed an alloy. The presence of nitrogen in large amounts obtained from PVP can easily be converted into N-doped carbon.

**Figure 13:**
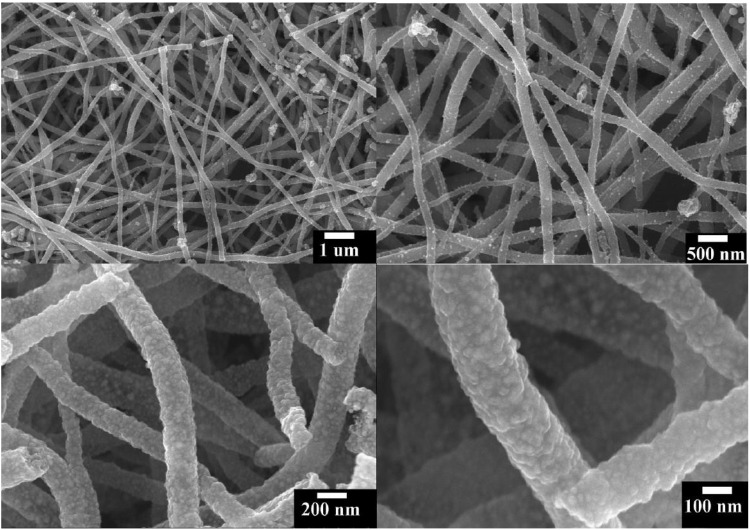
Field emission scanning electron microscope (FESEM) images at different magnifications for the obtained powder after calcinations of the electrospun NiAc/CoAc/PVA nanofiber mats in argon atmosphere at 800°C for 5 h [Reprinted with permission from Barakat et al. (2010), Copyright 2010, The Journal of Physical Chemistry C].

Magnetic anisotropic nanostructure has garnered great interest as it strongly influences the shape of hysteresis loop and, hence, controls the coercivity and remanence. Among magnetic nanostructures, Co-Ni has gained more attention since it is capable of overcoming the super magnetic edge and further improve the contrast in magnetic resonance imaging. Co-Ni bimetallic fine nanofibers were synthesized by the electrospinning technique (Barakat et al., 2010). The synthesis followed mixing of two metal precursors, nickel acetate tetrahydrate and cobalt acetate tetrahydrate, in an aqueous solution in a 1:1 ratio and then dissolved in poly(vinyl alcohol)/H_2_O solution. The solution was allowed to stir for 5 h at 52°C. A voltage of 20 kV was applied to the solution through a high voltage generator. The solution is present in the capillary with an optimized inclination angle. A ground iron drum immersed in polyethylene sheet used as a counter electrode. The synthesized nanofibers were further dried for 24 h and allowed to calcined at 800°C for 5 h in an argon atmosphere with a heating rate of 2.3°C/min. Figure 13 shows the FESEM image of synthesized Co-Ni nanofibers in different magnifications.

## Application of Anisotropic Bimetallic Nanostructure in Catalysis

A catalyst acts as the initiator to enhance the reaction rate by decreasing the threshold barrier, which helps to progress the reaction in less time and at a lower temperature and pressure. Basically, it decreases the activation energy to convert the reactant into the product faster. Furthermore, catalytic performance has been significantly developed with the advent of nanotechnology, which made it possible to use nanosized particles to catalyze various chemical reactions. Catalytic behavior absolutely depends on the shape, size, and morphology of the two metals. BNS have been extensively explored and are an interesting area of heterogeneous catalysis in many applications, especially for energy conversion and chemical transformations (Armbrster et al., 2010). Special interest in the synthesis of BNPs as catalyst is driven due to its alternation of electronic structure and generation of novel characteristics. When the second metal (guest metal) is added to the first metal (host metal) the combination of metals with its pristine property enhances catalytic activity, higher selectivity, and stability (Service, 2007). The combination of the guest metal with the host metal develops either: (a) an electronic effect or ligand effect, in which there is an alteration of electron properties or electronic arrangement in the active sites of the host metal due to an electron transfer from guest to host metals. The concept to estimate the influence of the guest metal over the host metal is the d-band center of host metal. (b) A geometric effect, when the guest metal atom coordinates with the host metal atom, it undergoes a different atomic arrangement generating new geometry with active sites over the newly generated surface of a bimetallic nanostructure (Siahrostami et al., 2013; Kim et al., 2014). A comparative study between the bimetallic single crystals of nanocatalysts alloy i.e., platinum–nickel and single crystals of pure platinum as well as platinum-carbon fuel cell catalysts in which bimetallic Pt_3_Ni possess different atomic arrangements in the crystalline lattice. The plane with the most tightly packed arrangement of atoms are more active in comparison to conventional and monometallic nanocatalysts. The activity of bimetallic Pt_3_Ni single-crystals increased to 10 and 90 folds with respect to single-crystal Pt and standard Pt/C materials. The theoretical model had predicted that the (111) arrangement decreased the electronic interaction among platinum atoms and oxides at the surface (Service, 2007). The catalytic performance of Pd can be improved by the rational synthesis of Pd-based BNS (Koenigsmann et al., 2012). Non-noble metals such as Co (Xu C. et al., 2013; Xu Y. et al., 2013), Cu (Xu et al., 2011), Ni (Maiyalagan and Scott, 2010), Sn (Ding et al., 2013), and Pd based bimetallic electrocatalysts show excellent catalytic activity and are cost effective. In Au-Pd the growth assembly and ligand effects in the nanostructures leads to extremely high activity (Zhang L. et al., 2016). Anisotropic nanostructures with sharp edges are highly reactive and show enhanced catalytic activity. Nanoparticles with an irregular shape lack such properties, resulting in their lower performance. But certain reactions need harsh conditions and pretreatments due to the instability of the morphology and form irregular shapes. The elevated reaction temperature upholds the rate of diffusion in the atoms and strengthens the particles to reorganize. Therefore, it is difficult to study the structural effect unless with a lack of stability in the morphology at high temperature. However, different structures have different properties which are responsible for various catalytic activities. Furthermore, the controlled synthesis of nanomaterials has offered an organized model to clarify the structural effect on the catalytic behavior (He et al., 2010). Wang and co reported that the synthesized bimetallic Au/Pd catalysts have a direct correlation between the structures of the catalysts and their catalytic behavior (Wang et al., 2008). Kuai and co have elucidated the excellent electrocatalytic property of Au-Pd alloy in the ORR where polycrystalline Au-Pd alloy showed enhanced catalytic performance compared to the core-shell (Kuai et al., 2012). Table 1 illustrates the diverse application of various BNS in the field of catalysis synthesized adapting different technique.

**Table 1:** Mentioned methods and BNS in different catalytic application.

**Material**	**Synthesis methods**	**Application in catalysis**	**References**
Nickel-Iron Phosphides Nanorods	Hydrothermal method	Hydrogen Evolution Reaction	Du et al., 2018
Pt-on-Pd Bimetallic Nanodendrites	Seed mediated growth strategy	Methanol oxidation reaction (MOR)	Kunz et al., 2017
Pd–Ir octapods and Nanocages	Galvanic replacement and co-reduction	Hydrazine decomposition	Liu et al., 2013
Pd crown-Au jewel Nanocluster	Galvanic replacement reaction	Aerobic glucose oxidation	Zhang H. et al., 2012
Au-Pd truncated hexoctahedral (THOH) shape	One-pot synthesis	Electrocatalytic performance for ethanol oxidation	Park et al., 2014
Dendritic Bimetallic Platinum–Nickel	One-pot synthesis	Oxygen Reduction Reaction	Eid et al., 2016
Nanocrystals Pd-Ce carbon	Electrospinning technique	Methanol fuel cells	Alvi and Akhtar, 2016
Nanofibers Nickel–Iron Oxide/Carbon Nanofibers	Electrospinning technique	Water oxidation electrocatalysis	Chen et al., 2016

### Bimetallic Nanostructure in the Field of Electrocatalysis

#### Hydrogen Evolution Reaction

Hydrogen Evolution Reaction (HER) is a half reaction of electrochemical water splitting and requires a proficient and stable electrocatalyst to reduce the overpotential for various applications (Tian et al., 2014; Yang et al., 2016). According to the literature, Pt/C is an excellent electrocatalyst for HER, however it is expensive with a low crystal content. An enormous effort has been made for the development of low-cost metal catalysts with phosphides doping (Cui et al., 2015). Ni-Fe phosphide nanorods exhibited electrocatalytic activity with a lesser overpotential of 243 mV (vs. RHE) at 20 mA.cm^−2^, smaller Tafel slope of 69.0 mVdec^−1^, and a higher exchange current density of 79.4 μA cm^−2^ in the HER within basic medium (Du et al., 2018). The synergetic effect developed between NiP_2_ and FeP enhanced the electrocatalytic activity of NiFeP bimetallic phosphides nanorods. Moreover, the anisotropic growth of NiFeP to form rod-like structures also exhibited a vital role for the enhancement of electrocatalytic performance.

Lu and co demonstrated the dependency over the crystal phases for the development of desired heterometallic nanostructures [i.e., Au–Ru nanowires (NWs)]. Ru nanorods can grow epitaxially to ~83.3% on the 4H phase of Au NWs while on fcc-twin structures the growth is restricted to ~16.7% (Lu et al., 2018). Since Ru is good candidate for HER, a comparative study of electrocatalytic activity for HER among Au–Ru NWs, 4H/fcc Au NWs, and Ru/C was carried out. Au–Ru NWs showed the best HER electrocatalytic activity with good stability till 10,000 cycles while 4H/fcc Au NWs exhibited no activity. With the overpotential of 50 mV, Au–Ru NWs attained a current density of 10 mA cm^−2^ which is less in comparison to Ru/C (116 mV). In addition to that, it exhibited a lower Tafel slope of 30.8 mV per decade while the Ru/C catalyst exhibited 47.8 mV per decade, which suggests Au–Ru NWs holds faster reaction kinetics in HER process. The greater electrocatalytic activity of bimetallic Au–Ru NWs resulted due to the following reasons: (1) One dimensional morphology of Au–Ru NWs facilitates the robust electron transfer throughout HER process. (2) The hierarchical arrangement of Au–Ru NWs generates plentiful active sites for HER. The 4H and fcc*-*twin morphology of Ru nanorods possess more surface atoms due to its concave and convex surfaces which provide additional benefits in HER activity (Fan et al., 2016). (3) Due to lattice strain and electron charge transfer between two different metal atoms, there is a change in the electronic band structure generating a favorable electronic band structure for its electrocatalytic performance (Wang et al., 2013; Wang H. et al., 2016).

#### Oxygen Reduction Reaction

Pt based bimetallic materials are foremost among all other materials for their superior electrocatalytic behavior in ORR for proton exchange membrane fuel cell (PEMFC) (Choi et al., 2016; O'hayre et al., 2016; Setzler et al., 2016).

**Figure 14:**
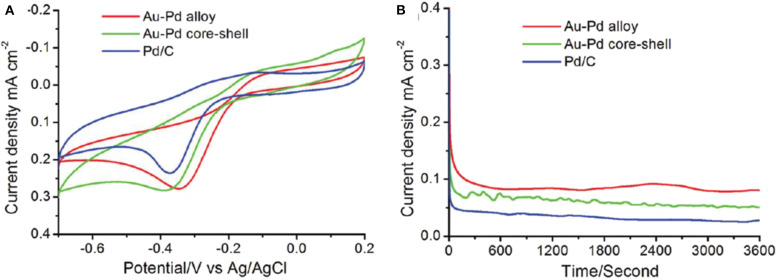
**(A)** CVs **(B)** and current-time curves of Au-Pd polycrystalline alloy (red line), core-shell nanostructures (green line), and Pd/C (blue line) electrocatalysts in O_2_-saturated 1 M KOH solution measured at room temperature, and the current-time curves are all recorded at the corresponding peak potential [Reprinted with permission from Kuai et al. (2012), Langmuir].

A comparative study in the electrocatalytic performance between an Au-Pd alloy and core shell nanostructure in the alkaline medium for ORR has been studied (Kuai et al., 2012). Figure 14A shows cyclic voltammograms (CVs) of an Au-Pd alloy (red line), core-shell (green line) nanostructures, and Pd/C catalyst (blue line) in an O_2_-saturated 1 M KOH solution. The corresponding catalyzed ORR performance is given in Table 2. The Au-Pd alloy nanostructures exhibited the finest electrocatalytic ORR activity. The Au-Pd polycrystalline alloy showed a current density around 1.2 times higher than the Pd/C catalyst is shown in Figure 14B. There is a simultaneous decrease in the overpotential of oxygen reduction. The alloy exhibited a positive shift in the peak potential of 44 and 34 mV with respect to the Au-Pd core-shell and Pd/C catalysts, respectively. Hence, the Au-Pd alloy nanostructures showed better ORR performance.

**Table 2:** The mentioned electrocatalysts and their corresponding catalyzing ORR performance [Reprinted with permission from Kuai et al. (2012), Langmuir].

**Sample**	**Peak potential/V**	**Peak current density/mA cm^**−2**^**	**Stable current density/mA cm^**−2**^**
**Electrocatalytic ORR performance**			
Au-Pd alloy	−0.342	0.281	0.080
Au-Pd core- shell	−0.386	0.285	0.051
Pd/C	−0.376	0.25	0.028

Urchin-like Pt-Ni BNS have shown superior ORR behavior both with and without any anion adsorption electrolytes, due to the synergistic effect developed from alloys. The synthesized Pt-Ni bimetallic showed 20.7 times better specific activity than monometallic Pt (Choi et al., 2016). Pt-Pd carbon supported nanodendrite catalysts were used for ORR performance and were compared with a commercial Pt/C catalyst (Wu et al., 2019a).

**Figure 15:**
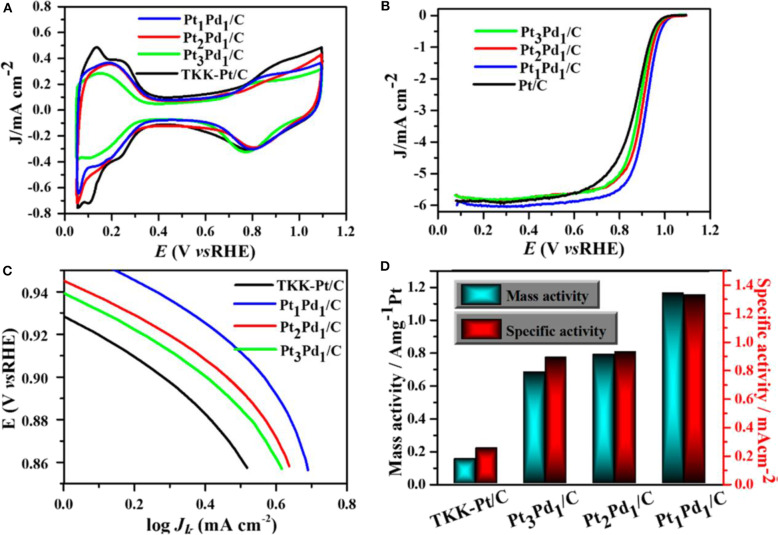
**(A)** Cyclic voltammograms recorded at room temperature in an N_2_-purged 0.1 M HClO_4_ solution with a sweep rate of 50 mV s^−1^. **(B)** ORR polarization curves recorded in an O_2_-saturated 0.1 M HClO_4_ solution with a sweep rate of 10 mV s^−1^ and a rotation rate of 1,600 rpm. **(C)** Tafel plots of four kinds of catalysts, and **(D)** histogram of specific mass activities at 0.9 V vs. RHE for four catalysts [Reprinted with permission from Wu et al. (2019b), ACS Sustainable Chemistry and Engineering].

A comparative study with three samples of different Pt loading in PtPd/C composites and commercial Pt/C catalysts of Pt loading in Pt_1_Pd_1_/C, Pt_2_Pd_1_/C, Pt_3_Pd_1_/C, and Pt/C were 8.3, 9.5, 12.6, and 24.3 μg.cm^−2^, respectively. The Pt_1_Pd_1_/C catalyst showed an excellent performance with good durability. The comparative study of CV among carbon-supported composites such as Pt_3_Pd_1_, Pt_2_Pd_1_, and Pt_1_Pd_1_with the commercial Pt/C catalyst is shown in Figure 15A. The CV study was carried out inside N_2_-purged 0.1 M HClO_4_ with a scan rate of 50 mV/s. An ORR polarization curve shows the half-wave potentials of different electrocatalysts were in the sort of commercial Pt/C (863 mV) < Pt_3_Pd_1_/C (884 mV) < Pt_2_Pd_1_/C (899 mV) < Pt_1_Pd_1_/C (916 mV) vs. a reversible hydrogen electrode (RHE) shown in Figure 15B. Tafel plots of ORR catalysts have been shown in Figure 15C in which all the catalyst followed similar Tafel slopes in common potential range and followed same reaction mechanism. Figure 15D shows histogram of specific mass activities at 0.9 V vs. RHE for four catalysts. The Pt_1_Pd_1_/C nanodendrite displayed the highest mass activity of 1.16 A ${\text{mg}}_{\text{Pt}}^{-1}$ and specific activity of 1.33 mA cm^−2^, which were 7.76- and 5.32-fold higher compared with the commercial Pt/C catalyst (0.15 A ${\text{mg}}_{\text{Pt}}^{-1}$ and 0.25 mA cm^−2^), respectively.

#### Oxygen Evolution Reaction

An amorphous Ni-Fe oxide/carbon nanofiber compound exhibited a high electrocatalytic response with superior stability in oxygen evolution reaction (OER) using 1M KOH electrolyte (Chen et al., 2016). The samples were prepared at different temperatures and studied by measuring the linear sweep voltammetry (LSV) curves that possessed maximum electrocatalytic activity for Ni_1_Fe_2_ material synthesized at 250°C and the activity order at different temperatures was as follows: 250 > 350 > 450 = 550°C. The LSV curve of Ni_1_Fe_2_-250 exhibited a sharp signal due to the oxidation of Ni^2+^ at 1.45 V (Trotochaud et al., 2014). When the potential was higher than 1.48 V, there was sudden rise in the oxidation current with the formation of bubbles over the electrode signaling the OER performance preceded. The slight decrease in the electrochemical surface areas (ECSA) of Ni_1_Fe_2_-250 to Ni_1_Fe_2_-350 and the significant decrease occurred subsequently for the samples synthesized at 450 and 550 °C. Ni_1_Fe_2_-250 was the largest ECSA and showed better OER performance, which helps the mass transport.

### Bimetallic Nanostructures in the Field of Photocatalysis

From previous discussion, it was observed that BNS provides novel characteristics by increasing the catalytic performance. In addition to that, photocatalysis has the potential ability to alter the localized surface plasmon resonance (LSPR) to acquire the desired performance. Plasmonic BNS have been extensively studied due to their assimilation of plasmonic function and catalytic effect. The massive and continuous demands from the industry are motivating researchers to develop efficient bimetallic photocatalysts. For example, Au-Cu triangular nanocrystal was used to catalyze the reduction of 4-nitrophenol to 4-aminophenol and the reaction was carried out under light irradiation as well as in the dark. It was found that the rate constant increased to 32 times when the reaction was conducted in the light in comparison to the dark (Hajfathalian et al., 2015). In addition, Pt-modified Au nanorods and triangular nanoprisms served as photocatalysts under visible and near-infrared light for hydrogen generation in water-methanol solutions (Zheng et al., 2014; Lou et al., 2016).

**Figure 16:**
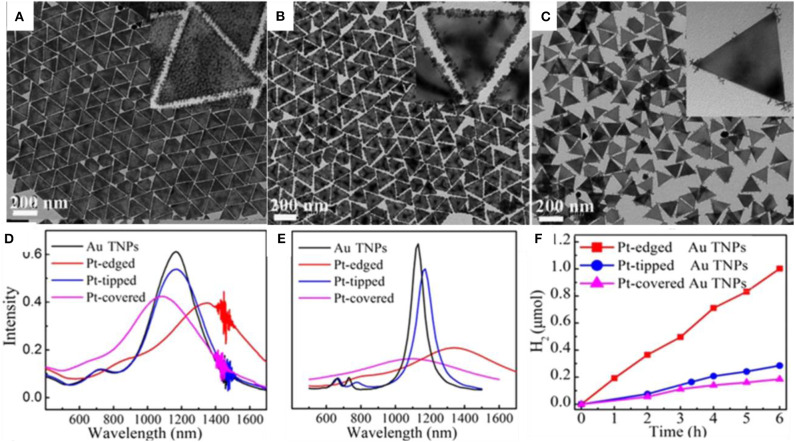
TEM images of **(A)** Pt-covered, **(B)** Pt edged, **(C)** Pt-tipped Au TNPs. Scale bar, 200 nm. **(D)** Visible-NIR extinction spectra **(E)** simulated extinction spectra of Au TNPs (black), Pt-covered (pink), Pt-edged (red), and Pt-tipped (blue) Au TNPs. In panel **(D)**, the noise around 1,500 nm is from surfactant of CTAC in the background. **(F)** H_2_ generation over Pt-covered (N), Pt-tipped (•), and Pt-edged (□) Au TNPs under visible-NIR light irradiation (>420 nm) [Reprinted with permission from Lou et al. (2016), ACS nano].

Using a modified seeded growth method, Lou and co successfully synthesized three different anisotropic nanostructures: Pt-covered, Pt-edged, and Pt-tipped Au triangular nanoprisms (TNPs) as shown in Figures 16A–C. Further, the overgrowth of Pt controlled over the Au surface with the optimal addition of H_2_PtCl_6_ and its photocatalytic dependency over hydrogen generation in water-methanol solutions (2:1 volume ratio) was studied under visible-NIR light irradiation. The rate of generation of H_2_ decreased in the order of Pt-edged, Pt tipped, and Pt-covered Au TNPs as shown in Figure 16F. The rate of hydrogen generation in Pt-edged Au TNPs was 0.167 μmol h^−1^, which was 5 and 3 times greater than Pt-covered (0.031 μmol h^−1^) and Pt-tipped Au TNPs (0.048 μmol h^−1^). The enhancement of H_2_ generation was well studied from surface plasmon resonance (SPR) in visible- NIR region as shown in Figures 16D,E. The SPR of different TNPs was performed using finite-difference-time-domain (FDTD). TNPs of Au poses different modes of SPR in visible-NIR regions i.e., in-plane dipole surface plasmon resonance (DSPR), multipole surface plasmon resonance (MSPR), and out-of-plane SPR (Tcherniak et al., 2011; Fang et al., 2012). The DSPR and MSPR band of Pt-edged Au TNPs showed red shifted with lesser intensity in comparison to Au TNPs followed by Pt tipped and Pt-covered Au TNPs, respectively, which confirmed the overgrowth of Pt on Au TNPs. Further, it suggests Pt is loaded with a stronger electric field and interaction with Au TNPs in comparison to other anisotropic structures, facilitating more hot electrons transfer among Au and Pt. Thus, it provides a capable charge separation and H_2_ generation of Pt-edged Au TNPs. Site-selective silica coating was used to block the overgrowth of the second metal Pd and allowed for preferential deposition at the end or side of synthesized Au nanorods. Au/SiO_2_/Pd nanobipyramids showed efficient plasmonic photocatalytic performance in the Suzuki coupling reaction.

### Oxidation Reaction

Rh, Ag, and Au are well-reported for their catalytic activity, but coupling these three metals is rarely reported. Wei and co grew RhAg on a template of Au nanorods with different structures and studied their catalytic properties in the oxidation of o-phenylenediamine (OPD) to 2,3-diaminophenazine (DAP). Bimetallic RhAg on Au nanorods with dumbbell-like structures were successfully synthesized (Ye et al., 2014). First, Au nanorods were synthesized and then the synthesized Au NR solution was mixed with a stabilizing agent CTAC, AgNO_3_, RhCl_3_, and reducing agent AA. With the same reaction protocol, brushy RhAg-coated Au nanorods and rod-like Au@Ag-Rh nanorattles were synthesized with the additional treatment of different concentrations of NaOH. The addition of NaOH affects the reaction pH and, hence, enhances the reducing capability. As a result, an increase in RhAg was observed on the surface of Au nanorods. Upon the addition of 0.41 mM NaOH, brushy nanorods formed with a length of 52.6 ± 5.2 nm and diameter of 29.0 ± 2.5 nm. With the increase in the concentration of NaOH to 1.66 mM, there was a formation of rod-like nanorattles, the core–shell Au@Ag nanorods covered with porous shell of Rh. The catalytic activity of the three different structures in the oxidation of o-phenylenediamine (OPD) to 2,3-diaminophenazine (DAP) were studied using UV-Vis spectroscopy. DAP has characteristic absorption band at 420 nm. It was found that brushy RhAg-coated gold nanorods showed higher catalytic performance in comparison to dumbbell-like RhAg-tipped gold nanorods and the rod-like Au@Ag–Rh nanorattles. The difference in the catalytic performance is due to the different structures possessing different surface energies and affecting active sites of Rh in the catalytic activity. Pd Rh nanoframes and nanoboxes have been reported for enhanced catalytic properties compared to the nanocubes (Ye et al., 2015). DAP shows absorption at 420 nm and OPD does not show any absorption (Cui et al., 2015). On conversion of OPD to DAP, there was a temporary rise in the absorption spectra using bimetallic Pd–Rh nanoboxes as the catalyst. Around 95% of OPD was transformed to DAP after 30 min with the rise in the absorption at 420 nm. The oxidation of OPDA to DAP using hollow and solid counterparts of nanoframes and nanoboxes have shown enhanced catalytic properties compared to the nanocubes.

## Summaries and Outlook

In the present review, previous and current evolution of the synthesis technique for anisotropic bimetallic nanomaterials and their applications in catalysis has been discussed. Each synthetic protocol has its own advantage and disadvantage and were designed based on applications that have been discussed. Transition or noble bimetallic nanocrystals and their synthesis and the formation of core shell and alloy with anisotropic nanostructures were given emphasis. Designing of the nanostructures and the arrangement of atoms exhibiting a strong relation over the surface of nanocrystals was also discussed. Special attention has been given for controlling the morphology, size, and shape of the nanostructures depending on their application. Additionally, many studies have been discussed, including the amalgamation of multiple elements significantly important for the advanced catalytic behavior (Choi et al., 2016; Du et al., 2018). Electronic arrangement in the BNS being the cause of synergy effect was explained in the various systems. The sequential reduction of gold, silver, palladium, and copper have been discussed in regards to the difference in the reduction potential between two metals that has largely been rationalized in the formation of core shell structures. Rate controlled SMCR reaction offers a strong and adaptable synthetic approach for synthesizing controlled anisotropic multimetallic nanostructures. Au-Pd bimetallic nanocrystals of high-index facets have demonstrated outstanding properties and exhibit their potential for wide applications. Apart from this, noble metal low-cost transition metals have also been given considerable interest for the synthesis of BNS. The GRR has marked a real breakthrough in the advancement of designing and synthesis of bimetallic nanomaterials. GRR was combined with the seed-mediated growth and the Kirkendall effect in order to achieve more complex BNS. Noble/non-noble BNS requires harsh reaction environments for one pot technique for the control of nucleation process and the kinetic growth of the two metals. The fact that the one pot technique avoids the prolonged separation technique and the removal of intermediate compounds to save time with a higher yield was explained with examples. BNS synthesized using hydrothermal protocol offers high purity, controlled morphology, uniformity, and fewer defects. Different temperatures in the hydrothermal technique provides diverse structures. The electrospinning technique is used for achieving extremely thin fibers of a few nanometers with high surface areas and pronounced physiochemical characteristics. In addition to morphology, the change in the precursor ratio in the mother solution exhibits wide tunability in the application. The synergy effect between two distinct atoms on a nanocrystal surface has been discussed widely with regards to their excellent catalytic activity, greater stability, and higher selectivity compared to monometallic analogs. ORR, HER, OER, and other catalysts have been tremendously accelerated by the BNS. Platinum, palladium monometallic, and bimetallic Au-Pd polycrystalline show higher electrocatalytic performance than Pd/C. Ni-Fe phosphides nanorods show better electrocatalytic performance with a smaller overpotential in comparison to Pt/C for HER.

## Author Contributions

PB and MB prepared the detailed synthesis part of bimetallic nanostructures. SS prepared the application in catalysis. MS and AS helped in the required modification and helpful discussion in this review.

## Conflict of Interest

The authors declare that the research was conducted in the absence of any commercial or financial relationships that could be construed as a potential conflict of interest.
